# Strategies for the implementation of clinical practice guidelines in public health: an overview of systematic reviews

**DOI:** 10.1186/s12961-022-00815-4

**Published:** 2022-01-24

**Authors:** Viviane C. Pereira, Sarah N. Silva, Viviane K. S. Carvalho, Fernando Zanghelini, Jorge O. M. Barreto

**Affiliations:** 1 Oswaldo Cruz Foundation, Brasília, Brazil; 2 Brazilian Ministry of Health, Brasília, Brazil

**Keywords:** Guidelines, Guideline implementation, Health system

## Abstract

**Background:**

As a source of readily available evidence, rigorously synthesized and interpreted by expert clinicians and methodologists, clinical guidelines are part of an evidence-based practice toolkit, which, transformed into practice recommendations, have the potential to improve both the process of care and patient outcomes. In Brazil, the process of development and updating of the clinical guidelines for the Brazilian Unified Health System (Sistema Único de Saúde, SUS) is already well systematized by the Ministry of Health. However, the implementation process of those guidelines has not yet been discussed and well structured. Therefore, the first step of this project and the primary objective of this study was to summarize the evidence on the effectiveness of strategies used to promote clinical practice guideline implementation and dissemination.

**Methods:**

This overview used systematic review methodology to locate and evaluate published systematic reviews regarding strategies for clinical practice guideline implementation and adhered to the PRISMA guidelines for systematic review (PRISMA).

**Results:**

This overview identified 36 systematic reviews regarding 30 strategies targeting healthcare organizations, healthcare providers and patients to promote guideline implementation. The most reported interventions were educational materials, educational meetings, reminders, academic detailing and audit and feedback. Care pathways—single intervention, educational meeting—single intervention, organizational culture, and audit and feedback—both strategies implemented in combination with others—were strategies categorized as generally effective from the systematic reviews. In the meta-analyses, when used alone, organizational culture, educational intervention and reminders proved to be effective in promoting physicians' adherence to the guidelines. When used in conjunction with other strategies, organizational culture also proved to be effective. For patient-related outcomes, education intervention showed effective results for disease target results at a short and long term.

**Conclusion:**

This overview provides a broad summary of the best evidence on guideline implementation. Even if the included literature highlights the various limitations related to the lack of standardization, the methodological quality of the studies, and especially the lack of conclusion about the superiority of one strategy over another, the summary of the results provided by this study provides information on strategies that have been most widely studied in the last few years and their effectiveness in the context in which they were applied. Therefore, this panorama can support strategy decision-making adequate for SUS and other health systems, seeking to positively impact on the appropriate use of guidelines, healthcare outcomes and the sustainability of the SUS.

**Supplementary Information:**

The online version contains supplementary material available at 10.1186/s12961-022-00815-4.

## Background

Clinical guidelines are defined as “systematically developed statements to assist practitioner and patient decisions about appropriate healthcare for specific clinical circumstances” [1]. As a source of readily available evidence, rigorously synthesized and interpreted by expert clinicians and methodologists, guidelines are part of an evidence-based practice toolkit which, transformed into practice recommendations, have the potential to improve both the process of care and patient outcomes [2]. For example, greater adherence to guidelines has been associated with reduced morbidity after appendectomy for complicated appendicitis, better and faster outcomes in patients with psychiatric disorders, better physical functioning outcomes, and less use of low back pain care [3–5].

However, although guidelines may be seen as important tools that support decision-making, in conjunction with clinical judgement and patient preference, there is still a lack of adherence to guidelines worldwide across different conditions and levels of care [6–8]. Studies from different countries have demonstrated suboptimal adherence to guidelines for low back pain in primary care, including the use of interventions with little or no benefit [9]. Among Australian nutritionists who provide clinical care to cancer patients, evidence indicates that only a third of the guidelines are routinely followed [10]. In Switzerland and Norway, a study found low overall adherence to current practice guidelines and high variation in the use of nutritional therapy in patients undergoing stem cell transplantation [11]. A study carried out in Norway showed low adherence of regular general practitioners to the palliative care guideline [12]. In the management of osteoarthritis, studies suggest that the main approaches recommended in the guidelines are underutilized and that the quality of care is inconsistent [13].

Numerous factors can influence the acceptance and use of guidelines, which may occur at the micro (individual behavioural, including clinicians and consumers), meso (organizational) or macro (context and system) level [14]. Some of these factors are intrinsic to the nature of newly recommended practice or technology itself, individual characteristics of healthcare professionals, and organizational capacity of health services to collect, adapt, share and apply evidence [15–17]. Other factors are intrinsic to guidelines; for example, when recommendations are not at all explicit, or they are distorted or ambiguous, due to conflict of interest, variable methodological quality, or being poorly written, they may be viewed as inapplicable to patients or as reducing clinician autonomy [18–20].

Thus, producing and providing high-quality guidelines is no guarantee that the recommendations will be implemented in healthcare practice, and therefore an active implementation strategy is necessary to encourage their uptake [21]. An iterative process consisting of several steps is recommended, including adapting guidelines to local context, identifying barriers to their use, selecting and implementing tailored interventions to promote guideline uptake, and monitoring and evaluating the associated outcomes and the sustainability of recommendations. Regardless of how guidelines are developed, what resources are required to support their implementation, or whether it is the responsibility of other individuals or organizations to implement them, detailed instructions for guideline implementation are needed [22, 23].

While the importance of turning knowledge into action and using available evidence to inform clinical practice is widely recognized, it still presents a challenge to most health services across different levels of government. In Brazil, the process of development and updating of the clinical guidelines for the Brazilian Unified Health System (Sistema Único de Saúde, SUS) is already well systematized by the Brazilian Ministry of Health. However, the process for implementing those guidelines has not yet been discussed and well structured. Therefore, a partnership project to elaborate a validated framework for the implementation of clinical guidelines to be used within SUS is being developed by the Ministry of Health and Oswaldo Cruz Foundation. The first step of this project is to develop a review of the scientific literature with the aim of providing an overview of the strategies used to promote guideline implementation and their effectiveness [24].

Numerous systematic reviews have synthesized data from primary studies on the effectiveness of strategies for implementing guidelines in several clinical areas including mental health [25, 26], arthritis [27], asthma [28] and cardiovascular disease [29, 30]. With the growth in the publication of systematic reviews, the strategy of grouping data from reviews in a single study has become a useful means for providing ample evidence to decision-makers in the healthcare field [31]. In this sense, some initiatives have been carried out to systematize review data on the subject in question. Chan et al., for example, compiled data from systematic reviews on four specific strategies (reminders, educational outreach visits, audit and feedback, and provider incentives), and the study by Cheung et al. evaluated the reminders in changing professional behaviour in clinical settings [32, 33].

However, we did not find comprehensive studies in the global literature that synthesized this topic without restrictions to certain clinical areas and specific interventions. In this context, the primary objective of this study was to summarize the evidence on the effectiveness of different strategies used to promote clinical practice guideline implementation. This overview will provide a broad summary of the best evidence on guideline implementation to support strategy decision-making adequate for each context (national, regional, local levels) and clinical area, thus seeking to positively impact on healthcare outcomes and on the sustainability of the SUS.

## Methods

This overview of systematic reviews was carried out in accordance with a protocol that was registered in the PROSPERO international prospective register of systematic reviews on 2 June 2017 (registration number: CRD42017065682). It was conducted following recommendations from the Cochrane Collaboration and reported using the Preferred Reporting Items for Systematic Reviews and Meta-Analyses (PRISMA) checklist [34].

### Inclusion criteria

Studies were selected based on the following criteria.

#### Types of studies

Systematic reviews that evaluated different strategies to promote clinical practice guideline implementation within a health system at the organizational, operational and individual levels (clinicians and patients) were included. Studies were selected regardless of the clinical area and focus of the intervention.

An overview of systematic reviews was considered the appropriate method to address this issue, as the literature search had identified relevant, recent systematic reviews with potential to cover a larger number of initiatives of clinical guideline implementation. Therefore, only systematic reviews were included.

Systematic review has been defined as “a review of a clearly formulated question that uses systematic and explicit methods to identify, select and critically appraise relevant studies, and to extract and analyse data from the studies included in the review” [35]. Considering this definition, studies with the following characteristics were classified as systematic reviews:

- a clear research question;
- eligibility criteria and description of the study selection process;
- description of the time period, terms and databases used in the search.

Overviews of systematic reviews were not eligible for inclusion.

#### Types of participants

Participants were considered in relation to the level of clinical guideline implementation in health systems: at the macro-level (international, national), meso-level (regional, healthcare organizations), and micro-level (healthcare professionals or teams).

#### Types of interventions

Systematic reviews addressing any strategy for clinical practice guideline implementation were eligible for inclusion in this overview.

#### Comparator

No restrictions were applied to the comparator.

#### Outcomes

The following question guided the selection of studies:

1. What is the effectiveness of strategies used to promote guideline implementation?

The primary outcomes of interest were strategies for clinical practice guideline implementation in a health system (organization, provider and patient levels).

### Literature search

The literature search was conducted using the following electronic databases: MEDLINE/PubMed, Centre for Reviews and Dissemination (CRD), the Cochrane Library, CINAHL (Cumulative Index to Nursing and Allied Health Literature), EMBASE, Web of Science, Scopus, Health Systems Evidence, Rx for Change (Canadian Agency for Drugs and Technologies in Health, CADTH) and Epistemonikos. The following databases were indicated in the overview protocol but they were not used: Guidelines International Network (GIN) website and International Initiative for Impact Evaluation (3ie) database, as well as Google and Google Scholar.

The basic search strategy combined search terms related to “clinical and therapeutic guidelines” (guidelines, clinical protocols, critical pathways, consensus and health planning guidelines) and “implementation of guidelines” (adherence, compliance, dissemination, accordance, concordance, adoption, barriers). The search strategies adapted for the electronic databases are presented in Additional file 1. The searches were carried out until 19 July 2017, and then updated until August 2019. There was no restriction on country, language or date of publication. Conference abstracts and studies that were not available in full text were excluded.

The terms were searched in the title and abstract, unless otherwise indicated in Additional file 1. The search results from the PubMed, Web of Science, Cochrane Library, Scopus, Epistemonikos, Embase and CINAHL databases were imported into Covidence reference management software for study selection, and duplicates were removed. As for the results from the other databases, an Excel spreadsheet was used for the study selection process.

### Screening and selection of studies

Titles and abstracts of the retrieved studies were screened by two independent reviewers (VP and FZ; update—VP and VC). Full-text assessment of potentially eligible studies was then independently undertaken for final selection. Disagreements regarding eligibility of studies were resolved by discussion and consensus, and when necessary, by a third reviewer. The screening process and results were reported according to the PRISMA statement.

### Data extraction

Results from the included studies were systematically extracted by one reviewer (VP) according to the predefined protocol, and summarized in a table of evidence using a data collection template in Excel. A second reviewer checked the extracted data.

The following information was extracted: year; authors; title; objective; country; number of studies identified; characteristics of the target population; clinical area, type of outcome evaluated, strategies for clinical practice guideline implementation and their effectiveness; conclusion, limitations of the review, evidence gaps, source of funding for the study.

Data were extracted from selected systematic reviews and meta-analyses; however, when information from reviews was insufficient, the primary studies were consulted.

### Methodological quality assessment

The methodological quality assessment using the AMSTAR 2 (A Measurement Tool to Assess Systematic Reviews 2) instrument [36] was conducted by two independent reviewers (VP and FZ; update—VP and VC). Disagreements were resolved by discussion and consensus.

### Data analysis

In the predefined protocol, data analysis was described only as a narrative synthesis. We subsequently refined this process even further. For systematic reviews, no meta-analysis of data was conducted. The results were reported as presented in the systematic reviews and meta-analyses. When the information was insufficient or unclear, we consulted the primary studies of each review. To do this, we recounted (i) all comparisons analysed in each study included in the review and (ii) the statistically positive results for each comparison studied. Each comparison was considered to be strategy A versus strategy B for each separate outcome (i.e. comparison of educational meeting effect associated with local opinion leader vs educational meeting only for outcome physician adherence). Based on the proportion of statistically positive results compared to the total analyses performed, efficacy was categorized as (1) generally effective (more than two thirds of the studies in a review showed positive effects), (2) mixed effects (one third to two thirds of the studies showed positive effects) or (3) generally ineffective (less than a third of the studies showed positive effects) [33]. In order to reduce bias in the interpretation of results obtained from a small number of evaluated comparisons, a cut-off was established of 10 or more comparisons evaluated to present the results of using the strategies.

Overlap analysis of studies included in each systematic review was performed to avoid duplication of effective results. In the case of duplication, we considered the results for the study included in the systematic review that presented more details regarding the strategy used to promote clinical practice guideline implementation. In cases of duplication of studies between systematic reviews selected from the first and second searches, we considered those included in systematic reviews from the first search.

## Results

### Selection of studies

**Fig. 1:**
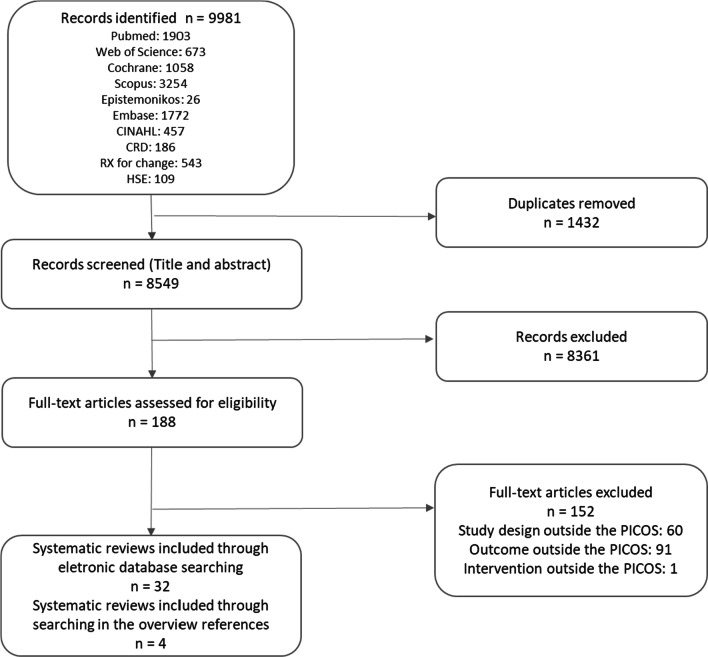
PRISMA flowchart of study selection. Source: own elaboration

Figure 1 presents a flowchart of the process used to identify relevant systematic reviews that were included. In total, 9981 articles were identified, of which 189 were selected for full-text reading, and then 32 met all inclusion criteria. Four systematic reviews identified in the references of excluded overviews were also included. The excluded studies along with reasons for exclusion are shown in Additional file 2.

### Characteristics of included studies

The systematic reviews included studies conducted in the following countries: United States (26 studies), United Kingdom (20 studies), Australia (14 studies), Netherlands (13 studies), Canada (12 studies), Germany (eight studies), France (six studies), Switzerland and Denmark (five studies each), Belgium, Thailand (four studies each), Iran, Brazil, Finland, Italy, Sweden, Norway (three studies each), Saudi Arabia, China, Singapore, New Zealand, Taiwan, Scotland, Spain, Mexico, Israel, Pakistan (two studies each), Ireland, Oceania, Argentina, Nepal, South Africa, Egypt, Oman, Japan, Korea, United Arab Emirates, Virgin Islands, South Africa, Georgia, Syria, China, Senegal, Mali, Benin, Malawi, Guatemala, India, Kenya and Zambia (one study each). There were also four studies conducted in a broader European setting (Table 1; Additional file 3).

**Table 1:** Characteristics of included studies

Study (article title)	Objective	Number of studies/clinical area	Country/setting	AMSTAR
“The effectiveness of interventions designed to increase the uptake of clinical practice guidelines and best practices among musculoskeletal professionals: a systematic review” [51]	To summarize and assess evidence about the effectiveness of knowledge translation interventions to improve the uptake and application of clinical practice guidelines and best practices for a wide range of musculoskeletal disorders and healthcare professionals	11 studies Musculoskeletal disorders	Netherlands, United Kingdom, Australia, United States, Switzerland, Ireland – Setting: NR	Low
“A review of quantitative studies of adherence to mental health clinical practice guidelines” [38]	To identify and review all published peer-reviewed reports providing quantitative information on rates of adherence to specific mental health guidelines	41 studies Mental health	United States, United Kingdom, Australia, Scotland, France, Canada – Primary care or other medical clinics, medical centres, nursing homes, acute medical ward, community-based sampling	Critically low
“Implementation of treatment guidelines for specialist mental health care” [26]	To examine the efficacy of guideline implementation strategies in improving process outcomes (performance of healthcare providers) and patient outcomes and the specific components of different guideline implementation strategies that could influence them	6 studies Schizophrenia-spectrum disorders	Denmark, German, United States, United Kingdom – Psychiatric units, hospitals	Critically low
“Assessing the effectiveness of strategies to implement clinical guidelines for the management of chronic diseases at primary care level in EU member states: a systematic review” [56]	To evaluate the effectiveness of strategies for implementing clinical guidelines for chronic disease management in primary care in EU member states	21 studies Chronic disease	EU member states: Finland, France, Germany, Italy, Spain, Sweden, United Kingdom, Denmark, Netherlands – Primary care level	Low
“Health professions digital education on clinical practice guidelines: a systematic review by digital health education collaboration” [63]	To evaluate the effectiveness of digital education in improving clinical practice guideline adoption	17 studies Several areas	Except for one study from an upper-middle-income country, all studies were from high-income countries, with 10 studies from the United States – Setting: NR	Critically low
“Evidence-based strategies for implementing guidelines in obstetrics: a systematic review” [43]	To determine which strategies have been effective for implementing clinical practice guidelines in obstetric care, and to identify barriers to change and facilitators in obstetrics	33 studies Obstetric care	Low-, middle- and high-resource settings – Obstetric care	Critically low
“Implementing a hospital guideline on pneumonia: a semi-quantitative review” [47]	To classify guideline implementation interventions used to improve treatment of community-acquired pneumonia and to quantify the impact of different interventions and their intensity of use on several processes of care and clinical and/or economic outcomes	27 studies Pneumonia	United States, United Kingdom, Canada, Australia – Hospital setting	Critically low
“The effectiveness of computerized clinical guidelines in the process of care: a systematic review” [66]	To determine the impact of computerized clinical guidelines on the process of care compared with non-computerized clinical guidelines	45 studies Acute and chronic	Europe, United States, Oceania – Academic and nonacademic centres	Critically low
“Information and communication technologies for the dissemination of clinical practice guidelines to health professionals: a systematic review” [61]	To identify studies on the perceived usability and practice behaviour change among health professionals with regard to information and communication technologies for the dissemination of clinical practice guidelines	21 studies NR	United States, Canada, Europe, and one international study – Setting: NR	Low
“A systematic review of the implementation and impact of asthma protocols” [41]	To determine the most widely used method of guideline implementation (paper, computer-generated or computerized) reported in the literature, which methods significantly improved clinical care, and the factors most commonly associated with successful and sustainable implementation of asthma guidelines	104 studies Asthma	United States, United Kingdom, Canada, Australia, Netherlands, Singapore, New Zealand, Brazil, Saudi Arabia, Germany, France, Oman, Switzerland, Italy, Iran, Japan, Taiwan, Korea, Thailand and United Arab Emirates – Outpatients, emergency department and inpatients, patients in other settings	Critically low
“Implementing guidelines in nursing homes: a systematic review” [53]	To systematically review the effects of interventions for improving the implementation of guidelines in nursing homes	5 studies Several areas	United States, Germany, Netherlands, Australia, Belgium – Nursing homes	Moderate
“Evidence-based guideline implementation in low- and middle-income countries: lessons for mental health care” [25]	To investigate studies on the effectiveness of evidence-based clinical practice guideline implementation across physical and mental healthcare in order to inform the provision of mental healthcare in low- and middle-income countries, and to identify transferable lessons from other noncommunicable diseases to mental health	6 studies Mental healthcare and physical health	Brazil, China, Thailand, Nepal, South Africa and Egypt – General hospitals in urban areas – Primary healthcare centres	Low
“Health professionals’ adherence to stroke clinical guidelines: a review of the literature” [44]	To review the literature on adherence to clinical guidelines including the impact of adherence on achieving quality indicators for stroke care, and to identify actual and perceived facilitators and barriers with respect to adherence to best practice	27 studies Stroke care	United States, Australia, United Kingdom, Scotland – National, regional, local and clinical institution level	Critically low
“Tools developed and disseminated by guideline producers to promote the uptake of their guidelines” [54]	To evaluate the effectiveness of implementation tools developed and disseminated by guideline producers, which accompany or follow the publication of a clinical practice guideline, to promote uptake. Secondarily, to determine which approaches to guideline implementation are most effective	4 studies Several areas	Netherlands, France, United States and Canada – Private physiotherapy practices, hospitals, family practice	Low
“Effectiveness and efficiency of guideline dissemination and implementation strategies” [58]	(1) To perform a systematic review of the effectiveness and costs of different guideline development, dissemination and implementation strategies (2) To estimate the resource implications of different development, dissemination and implementation strategies (3) To develop a framework for determining when it is efficient to develop and introduce clinical guidelines based upon the potential costs and benefits of the targeted clinical activity and the effectiveness and costs of guideline development and introduction	235 studies NR	Most in United States, United Kingdom, Canada, Australia and Netherlands – Primary care, inpatient settings and generalist outpatient or ambulatory care settings, mixed settings, nursing homes or long-term care facilities, emergency departments, specialist outpatient care and a military medical clinic	Low
“Implementing practice guidelines for appropriate antimicrobial usage: a systematic review” [50]	To identify the best methods for changing prescribing practices and to facilitate better implementation of these guidelines	40 studies Antimicrobial usage	NR	Critically low
“A systematic review of the outcomes of educational interventions relevant to nurses with simultaneous strategies for guideline implementation” [77]	To systematically review the literature regarding outcomes of educational interventions relevant to nurses with respect to guideline implementation	13 studies Several areas	United States, Australia, Iran, Singapore – Nursing units	Critically low
“Guideline implementation in allied health professions: a systematic review of the literature” [57]	To evaluate the effects of the introduction of clinical guidelines for allied health professionals, and the effectiveness of the guideline dissemination and implementation strategies used	14 studies NR	United States, Australia, Netherlands, United Kingdom – Clinical institution level, provider level	Critically low
“Effectiveness of electronic guideline-based implementation systems in ambulatory care settings – a systematic review” [64]	To perform a systematic and comprehensive search of the literature for studies that evaluated the effectiveness of computer-based systems for guideline implementation in ambulatory care settings, with the multidimensionality of the guideline (the guideline had to consist of several aspects or steps) and real-time interaction with the system during consultation as important inclusion criteria	27 studies Chronic and acute diseases and tobacco use cessation	United States, four in the United Kingdom, five in the Netherlands, two in Norway, one in France, and one in Finland – Ambulatory care, of which four were performed in the emergency department	Low
“A systematic review of implementation strategies to deliver guidelines on obstetric care practice in low- and middle-income countries” [42]	To evaluate whether strategies for promoting the use of guidelines could improve obstetric practices in low- and middle-income countries	9 studies Obstetric care	Georgia, Syria, China and Pakistan, Senegal, Mali, Benin, Malawi, Mexico, Thailand, Argentina, Guatemala, India, Pakistan, Kenya and Zambia – Health centres and hospitals, health facilities	Low
“Interventions to improve adherence to cardiovascular disease guidelines: a systematic review” [29]	To synthesize evidence on the effectiveness of interventions targeting healthcare providers to improve adherence to cardiovascular disease guidelines and patient outcomes	38 studies Cardiovascular	United States, Netherlands, Italy, England, and Norway, Canada, South Africa, Brazil, Asia–Pacific area and Virgin Islands – Setting: NR	Low
“Implementation strategies for guidelines at ICUs: a systematic review” [49]	To conduct a systematic review on implementation strategies for clinical practice guidelines in the intensive care unit, in order to assist critical care practitioners in the use of implementation strategies for clinical practice guidelines in the ICU	8 studies Critical care	Canada, Australia, England and Taiwan, and United States – Adult and neonatal ICUs	Critically low
“Use of theory to plan or evaluate guideline implementation among physicians: a scoping review” [65]	To summarize current research in the field of guideline implementation to determine whether and how theory has been used to plan or evaluate the implementation and use of guidelines among physicians, as frequent target users of guidelines	42 studies Several areas	United States, United Kingdom, Australia, Netherlands, Canada, Iran, Belgium, Germany and Saudi Arabia – Setting: NR	Critically low
“Educational interventions for implementation of arthritis clinical practice guidelines in primary care: effects on health professional behavior” [27]	To evaluate the influence of educational programmes to implement clinical practice guidelines for osteoarthritis and rheumatoid arthritis in primary care	7 studies Osteoarthritis and rheumatoid arthritis	Country: NR – Primary care environment	Critically low
“Improving adherence to guidelines for the diagnosis and management of pelvic inflammatory disease: a systematic review” [46]	To examine the strategies that may improve adherence to guidelines for pelvic inflammatory disease diagnosis and management	3 studies Pelvic inflammatory disease	United States – Hospital and outpatient facilities	Critically low
“Systematic review of practice guideline dissemination and implementation strategies for healthcare teams and team-based practice” [60]	To synthesize the literature relevant to guideline dissemination and implementation strategies for healthcare teams and team-based practice	89 studies Not reported	United States and the United Kingdom, Canada, Australia – Setting: NR	Critically low
“Enhancing prescribing of guideline-recommended medications for ischaemic heart diseases: a systematic review and meta-analysis of interventions targeted at healthcare professionals” [39]	To evaluate whether interventions targeted at healthcare professionals are effective in enhancing prescribing and health outcomes in patients with ischaemic heart diseases	13 studies Cardiology	North America and Europe – Setting: NR	Low
“Interventions to modify health care [provider adherence to asthma guidelines: a systematic review” [28]	To determine whether interventions targeting healthcare providers improve adherence to asthma care guidelines and thereby improve outcomes. Healthcare process outcomes such as patients receiving appropriate treatment, and clinical outcomes such as hospitalizations were considered	68 studies Asthma	Country: NR – Clinical institution level, provider level	Critically low
“Effectiveness of implementation interventions in improving physician adherence to guideline recommendations in heart failure: a systematic review” [40]	To examine the effectiveness of implementing interventions in increasing physician adherence to the specified heart failure guideline recommendations. Secondarily, to assess the effect of implementation interventions on clinical outcomes, and to identify process and contextual factors that influence implementation success	38 studies Cardiology	United States, Europe and Australia – Inpatient settings, outpatient settings and mixed settings	Low
“Effect of clinical guidelines in nursing, midwifery, and the therapies: a systematic review of evaluations” [59]	(1) To identify evaluations in any setting of clinical practice guidelines and related dissemination and implementation strategies in nursing, midwifery, health visiting and other professions allied to medicine, including podiatry, speech and language therapy, physiotherapy, occupational therapy, dietetics, clinical psychology, pharmacy and radiography (2) To estimate the effectiveness and cost-effectiveness of clinical practice guidelines for promoting improved professional practice and enhanced patient outcomes in nursing and professions allied to medicine	18 studies Pneumococcal and influenza vaccinations, use and follow-up of medications, urinary catheter care	Country: NR – Ambulatory medical practices	Critically low
“Implementation of pressure ulcer guidelines: what constitutes a successful strategy?” [48]	To identify studies on the effectiveness of strategies in implementing evidence-based guidelines and recommendations for the prevention and/or management of pressure ulcers and the characteristics associated with sustainable implementation of these strategies	20 studies Pressure ulcer	United States, German, United Kingdom, Australia – Hospitals, nursing homes, community care centres, residential homes, universities, tertiary care hospital, long-term care setting, regional healthcare system	Critically low
“Strategies for guideline implementation in primary care focusing on patients with cardiovascular disease: a systematic review” [30]	To compare different implementation strategies for guidelines targeting primary or secondary prevention and treatment of cardiovascular diseases	52 studies Cardiovascular disease	Canada, United States, European countries, Israel, Pakistan and Taiwan – Setting: NR	Critically low
“Multifaceted strategies may increase implementation of physiotherapy clinical guidelines: a systematic review” [45]	To determine the effectiveness of strategies to increase the implementation of physiotherapy clinical guidelines	3 studies Physiotherapy clinical	Australia, Netherlands, United Kingdom – Institutional and provider level (physiotherapists)	Critically low
“Effectiveness of implementation strategies for clinical guidelines to community pharmacy: a systematic review” [55]	To synthesize the literature on the implementation of clinical guidelines in community pharmacy	22 studies Several areas	Australia, United States United Kingdom, Netherlands, Belgium, Canada, Finland, Germany and Switzerland – Community pharmacy setting	Low
“Effects of implementation of psychiatric guidelines on provider performance and patient outcome: systematic review” [37]	To summarize the evidence pertaining to the benefits of mental health guidelines with respect to specific implementation strategies	18 studies Psychiatric	United States, United Kingdom, Denmark and Canada – Primary care, general hospital, specialist mental healthcare settings, mental health clinicians of a managed behavioural healthcare organization	Critically low
“Implementing guidelines and innovations in general practice: which interventions are effective?” [62]	To evaluate the effectiveness of interventions in promoting the implementation of guidelines and adoption of innovations in general practice	143 studies NR	United States, United Kingdom, Canada and several other countries – Setting: NR	Critically low

*EU* European Union, *ICU* intensive care unit, *NR* not reported

The systematic reviews evaluated strategies for guideline implementation at various levels of health services, including inpatient and outpatient settings, primary and secondary care settings, private clinics, community health clinics, nursing homes, academic institutions, emergency services and intensive care units.

As for the clinical areas covered, four systematic reviews evaluated strategies for guideline implementation and dissemination related to physical and mental healthcare [25, 26, 37, 38], two related to cardiovascular diseases [29, 30, 39, 40], asthma [28, 41] and obstetrics [42, 43], and one related to stroke [44], physical therapy [45], pelvic inflammatory disease [46], osteoarthritis and rheumatoid arthritis [27], pneumonia [47], pressure ulcers [48], intensive care units [49], prescription practices [50] and musculoskeletal disorders [51]. Some systematic reviews evaluated guidelines related to several clinical areas [52–66].

The methodological quality of the included systematic reviews was assessed using the AMSTAR 2 tool [36], which consists of 16 items. According to this assessment, over the past decade, systematic reviews have provided more information on methods and parameters used in the analyses. One systematic review showed moderate, 12 low and 23 critically low methodological quality. The low rating was due to failure in meeting AMSTAR 2 criteria on the following critical domains: no justification for excluding individual studies (80%), no protocol registered before commencement of the review (75%) and no consideration of risk of bias when interpreting results from the review (47%) (Table 1; Additional file 3).

### Strategies to promote clinical practice guideline implementation

The strategies reported in the systematic reviews were classified according to the Cochrane Effective Practice and Organisation of Care (EPOC) taxonomy of health interventions [67], and, when the strategy was not found in this taxonomy, we used the definition of systematic review of Grimshaw et al. [58]. Thirty strategies targeting healthcare organizations (*n* = 6), community (*n* = 1), health professionals (*n* = 21) and patients (*n* = 2) to promote guideline implementation were reported. Table 2 presents the strategies and their definitions.

**Table 2:** Strategies for clinical practice guideline implementation and their definitions

Category	Strategy	Code	Definition
Coordination of care and management of care processes	Care pathways	CAP	Aim to link evidence to practice for specific health conditions and local arrangements for delivering care [67]
Coordination of care and management of care processes	Case management	CAM	Introduction, modification or removal of strategies to improve the coordination and continuity of delivery of services, i.e. improving the management of one “case” (patient) [67]
Coordination of care and management of care processes	Clinical multidisciplinary teams	CMT	Creation of a new team of health professionals of different disciplines or additions of new members to the team who work together to care for patients [58, 67]
Coordination of care and management of care processes	Communication between providers	CBP	Systems or strategies for improving the communication between healthcare providers, for example systems to improve immunization coverage [67]
Coordination of care and management of care processes	Continuity of care	COC	Interventions to reduce fragmented care and undesirable consequences of fragmented care, for example by ensuring the responsibility of care is passed from one facility to another so the patient perceives that their needs and circumstances are known to the provider [67]
Information and communication technology	Information and communication technology	ICT	ICT used by healthcare organizations to manage the delivery of healthcare, and to deliver healthcare [67]
Changes to the healthcare environment	Structural intervention	SI	Changes to the setting/site of service delivery, physical structure, facilities and equipment, and medical records systems, among others [58]
Authority and accountability for health policies	Community mobilization	COM	Processes that enable people to organize among themselves [67]
Interventions targeted at healthcare workers	Academic detailing	AD	Personal visits by a trained person to health workers in their own settings, to provide information with the aim of changing practice [67]
Interventions targeted at healthcare workers	Audit and feedback	AF	A summary of health workers’ performance over a specified period of time, given to them in a written, electronic or verbal format. The summary may include recommendations for clinical action [67]
Interventions targeted at healthcare workers	Communities of practice	CP	Groups of people with a common interest who deepen their knowledge and expertise in this area by interacting on an ongoing basis [67]
Interventions targeted at healthcare workers	Continuous quality improvement	CQI	An iterative process to review and improve care that includes involvement of healthcare teams, analysis of a process or system, a structured process improvement method or problem-solving approach, and use of data analysis to assess changes [67]
Interventions targeted at healthcare workers	Educational games	EG	The use of games as an educational strategy to improve standards of care [67]
Interventions targeted at healthcare workers	Educational materials	EMA	Distribution of educational materials to individuals or groups, to support clinical care, i.e. any intervention in which knowledge is distributed [67] Distribution of published or printed recommendations for clinical care, including clinical practice guidelines, audiovisual materials and electronic publications. The materials may have been delivered personally or through mass mailings [58]
Interventions targeted at healthcare workers	Educational meetings	EME	Courses, workshops, conferences or other educational meetings [67]
Interventions targeted at healthcare workers	Local consensus processes	LCP	Formal or informal local consensus processes, for example agreeing on a clinical protocol to manage a patient group, adapting a guideline for a local health system or promoting the implementation of guidelines [67]
Interventions targeted at healthcare workers	Local opinion leaders	LOL	The identification and use of identifiable local opinion leaders to promote good clinical practice [67]
Interventions targeted at healthcare workers	Monitoring the performance of the delivery of healthcare	MP	Monitoring of health services by individuals or healthcare organizations, for example by comparing with an external standard [67]
Interventions targeted at healthcare workers	Patient-mediated Intervention	PMI	Any intervention aimed at changing the performance of healthcare professionals through interactions with patients, or information provided by or to patients [67]
Interventions targeted at healthcare workers	Reminders	RE	Manual or computerized interventions that prompt health workers to perform an action during a consultation with a patient, for example computer decision support systems [67]
Interventions targeted at healthcare workers	Tailored interventions	TI	Interventions to change practice that are selected based on an assessment of barriers to change, for example through interviews or surveys [67]
Interventions targeted at healthcare organizations	Organizational culture	ORG	Strategies to change organizational culture [67]
Interventions targeted at healthcare organizations	Financial interventions	FI	Targeted financial incentives for health professionals and healthcare organizations [67]
Interventions targeted at healthcare workers	Educational intervention	EI	Education-focused intervention [29]
NA	Patient incentives	PIC	Patient received direct or indirect financial reward or benefit for a specific action or to encourage them to do a specific action [58]
NA	Patient-directed interventions	PI	Interventions aimed at qualifying patients for self-care and for decision-making [46]
NA	Administrative restriction	AR	Administrative restrictions related to prescriptions [37]
NA	Marketing	MKT	Approaches that businesses would normally use to encourage people to use their materials [60]
NA	Mass media	MM	Varied use of communication that reached great numbers of people including television, radio, newspapers, posters, leaflets and booklets, alone or in conjunction with other interventions; targeted at the population level [67]
NA	Practice support	PS	Available professional to support the clinical practice or directly to the patient [26]

*NA* strategies not classified by the EPOC

Additionally, the strategies were classified according to the outcomes: process-, patient- and health professional-related outcomes, economic outcomes and nonspecific outcomes. In regard to single or multifaceted interventions, most outcomes were related to process, followed by patients and professionals. The most frequently reported strategies were educational materials, educational meetings, reminders, auditing and feedback, and academic detailing.

### Effectiveness of the clinical practice guideline implementation strategies

Information on the effectiveness of clinical practice guideline implementation strategies was collected by considering the number of statistically significant positive results from each comparison analysed in the systematic reviews. The percentages of effective results in relation to the total analyses performed for each strategy were categorized as generally effective, mixed effects and generally ineffective. As described in “Methods”, we only present the results of strategies with 10 or more comparisons analysed (Table 3). The results of all strategies are presented in Additional file 4.

**Table 3:** Effectiveness of guideline implementation strategies from systematic reviews by outcome

	Outcome							
Strategy	Process		Professional		Patient		Economic	References
	SS	MS	SS	MS	SS	MS	MS	
RE	⋄⋄	⋄		⋄⋄	⋄	⋄	⋄	[25–28, 37–44, 47–51, 54–59, 61, 62, 64–66, 77]
EMA	⋄	⋄	⋄⋄	⋄⋄	⋄⋄	⋄	⋄	[25–28, 37–40, 42–51, 53–56, 58–62, 65, 77]
EME	⋄⋄	⋄	⋄⋄⋄	⋄⋄	⋄	⋄	⋄	[25–28, 37–40, 42–45, 47–51, 53–56, 58, 59, 61, 62, 65, 77]
AF	⋄⋄	⋄⋄		⋄⋄⋄		⋄		[25, 27, 28, 37–40, 42–44, 48–50, 53, 55, 56, 58, 60–62, 77]
CMT	⋄	⋄				⋄⋄		[39, 40, 44, 47–49, 53, 77]
LOL	⋄⋄	⋄⋄				⋄		[25, 38–40, 42–45, 49, 50, 58, 59, 65]
CAP	⋄⋄⋄				⋄			[25, 38, 40, 43, 47, 50]
PS	⋄⋄	⋄⋄				⋄		[26–28, 37, 38, 40, 43, 44, 49, 50, 65]
AD		⋄				⋄	⋄	[25–27, 37–39, 42–45, 47, 49–51, 53–56, 58, 61, 65]
FI		⋄⋄						[40, 55, 57, 58, 65]
PI		⋄				⋄		[37, 38, 43, 46, 58, 59, 62]
COC		⋄						[40, 43]
ORG		⋄		⋄⋄⋄		⋄		[28, 39, 43, 44, 57–59, 62, 77]
SI		⋄				⋄		[40, 44, 47–49, 56, 58]
MP		⋄⋄						[25, 40, 43, 44, 48, 50, 51]
PMI		⋄				⋄		[47, 58]
COM						⋄		[42]
LCP		⋄				⋄		[42, 43, 47, 53, 58]
ICT		⋄⋄						[29, 37, 44, 50, 55, 61]

*CAP* care pathways, *CMT* clinical multidisciplinary teams, *COC* continuity of care, *COM* community mobilization, *ICT* information and communication technology, *SI* structural intervention, *AD* academic detailing, *AF* audit and feedback, *EMA* educational materials, *EME* educational meetings, *LCP* local consensus processes, *LOL* local opinion leaders, *MP* monitoring the performance of the delivery of healthcare, *PMI* patient-mediated intervention, *RE* reminders, *ORG* organizational culture, *FI* financial interventions, *PI* patient-directed interventions, *PS* practice support, *SS* single strategy, *MS* multifaceted strategy

The other strategies did not present ≥ 10 evaluated comparisons and, therefore, the results are presented in Additional file 4

**⋄⋄⋄**Generally effective (more than two thirds of comparisons in a review demonstrated statistically positive effects)

**⋄⋄**Mixed effects (one third to two thirds of studies demonstrated statistically positive effects)

**⋄**Generally ineffective (fewer than one third of studies demonstrated statistically positive effects)

Most process-related outcomes evaluated how guideline implementation strategies affected requests for examinations, prescription of medications and performance of procedures, and whether they were in accordance with the guidelines. For these outcomes, 628 and 1814 analyses of strategies implemented alone and in combination with others, respectively, were carried out (Table 3).

In the case of single interventions, care pathway was the only generally effective categorized strategy. Reminders, educational meetings, audit feedback, local opinion leaders and practice support were classified as strategies yielding mixed effects. In the evaluation of multifaceted interventions, none reached the percentage of results to be categorized as generally effective (Table 3).

Health professional-related outcomes evaluated the changes in professionals’ knowledge, attitudes, self-reported practice and self-confidence in using, satisfaction in following, and willingness to follow guidelines. A small number of analyses were performed for these outcomes, 39 for strategies implemented alone and 150 for multifaceted interventions (Table 3).

Educational materials and educational meetings were the most commonly reported strategies when implemented alone, the latter being classified as generally effective, and the former as having mixed effects. In the evaluation of multifaceted interventions, changes in organizational culture and the audit and feedback strategy were classified as generally effective, while educational materials and educational meetings and reminders showed mixed results for the outcomes related to health professionals (Table 3).

Patient-related outcomes addressed clinical information, quality of life and patient satisfaction with care received. For these outcomes, 113 and 752 analyses of strategies implemented alone and in combination with others, respectively, were carried out. When used as single or multifaceted strategies, no intervention was considered generally effective (Table 3).

A small number of studies evaluated the effectiveness of guideline implementation strategies related to economic outcomes (eight analyses for single interventions, and 90 analyses for multifaceted interventions), none of which proved effective.

Two meta-analyses were included in this study. In total, eight strategies were evaluated for outcomes related to processes and patients [29, 30]. When used alone, organizational culture, educational intervention and reminders proved to be effective in promoting physicians' adherence to the guidelines [30]. In patient-directed interventions, patient education was effective, and promotion of patient self-management showed a statistically nonsignificant small benefit for this outcome [30]. Still focusing on physician adherence, when used in conjunction with other strategies (multifaceted strategies), organizational culture proved to be effective, education intervention showed mixed effects (one meta-analysis with effective results and one meta-analysis without statistical difference), and patient-directed reminders, educational meetings, academic detailing and information and communication technology presented results without statistical significance [29, 30] (Table 4).

**Table 4:** Effectiveness of guideline implementation strategies from meta-analysis by outcome

Outcome Strategy	Process				Patient	
	Single strategy		Multifaceted strategy		Multifaceted strategy	
	Significant positive result	No statistically significant difference	Significant positive result	No statistically significant difference	Significant positive result	No statistically significant difference
Audit and feedback	-	–	–	– Adherence outcome/long-term (6 studies) [29] – Adherence outcome (4 studies)^a^ [29] – Adherence outcome (4 studies)^a^ [29] – Physician adherence (12 studies) [30]	–	– Disease target results in the long term (3 studies) [29]
Organizational culture	– Physician adherence (14 studies) [30]	–	– Physician adherence (17 studies) [30]	–	–	–
Educational interventions	– Physician adherence (15 studies) [30]	–	– Physician adherence (26 studies) [30]	– Adherence outcome/short-term (6 studies) [29] – Adherence outcome/long-term (8 studies) [29] – Adherence outcome (4 studies) [29]	– Disease target results in the short term (6 studies) [29] – Disease target results in the long term (5 studies) [29]	– Mortality in the short term (3 studies) [29] – Mortality in the long term (4 studies) [29] – Hospitalizations in the long term (4 studies) [29]
Patient-directed interventions	– Physician adherence (5 studies)^a^ [30]	– Physician adherence (5 studies)^a^ [30]	–	– Physician adherence(14 studies)^a^ [30] – Physician adherence (15 studies)^a^ [30]	–	–
Reminders	– Physician adherence (15 studies) [30]	–	–	– Physician adherence (22 studies) [30] – Adherence outcome/long-term (6 studies) [29] – Adherence outcome (4 studies) [29]	–	– Disease target results in the long term (3 studies) [29]
Educational meetings	–	–	–	– Adherence outcome/long-term (6 studies) [29] – Adherence outcome (4 studies) [29]	–	– Disease target results in the long term (3 studies) [29]
Information and communication technology	–	–	–	– Adherence outcome/long-term (6 studies) [29] – Adherence outcome (4 studies) [29]	–	– Disease target results in the long term (3 studies) [29]
Academic detailing	–	–	–	Adherence outcome/long-term (6 studies) [29] Adherence outcome (4 studies) [29]	–	– Disease target results in the long term (3 studies) [29]

^a^Different outcomes related to physician adherence

For patient-related outcomes, educational intervention showed effective results for disease targets in the short and long term, and with no difference for mortality and hospitalization. The other strategies (audit and feedback, reminders, educational meetings, information and communication technology, and academic detailing) did not show positive statistical results [6]. It should be noted that educational interventions are extremely heterogeneous strategies without standardization of the elements that they comprise, and they may range from general instructions to digital education (Table 4).

## Discussion

The objective of this study was to summarize the evidence on the effectiveness of different strategies used to promote clinical practice guideline implementation and dissemination. For this purpose, we synthesized the results of 36 systematic reviews on 30 strategies for guideline implementation. The scope of our study calls for caution in interpreting the effectiveness results, as no meta-analysis was performed, and the data were extracted from heterogeneous studies with different designs, clinical areas, contexts, intervention composition and outcomes. Thus, this data compilation can be useful as a map of the available evidence on guideline implementation strategies, on which clippings can be made according to the intended outcomes and the implementation context.

The strategies with the greatest volume of comparisons rated were educational materials, educational meetings, reminders, audit and feedback, and academic detailing. For outcomes related to processes assessed in systematic reviews, the only intervention categorized as generally effective when used alone was care pathways. Still, in the evaluation of these outcomes, the result of one of the included meta-analyses estimated that, when used alone, organizational culture, educational intervention, reminders and patient education were effective in promoting physicians' adherence to the guidelines. For multifaceted interventions, only organizational culture was effective.

Regarding the outcomes assessed in health professionals, educational meetings, used alone, and organizational culture and audit and feedback, both used in association with other strategies, were categorized as being generally effective with the data collected from systematic reviews. In evaluating the results of patients, systematic reviews did not present strategies categorized as generally effective; however, in one of the meta-analyses, educational interventions were effective for disease target results in the short and long term [29]. It should be noted that educational interventions are extremely heterogeneous strategies without standardization of the elements that they comprise, and they may range from general instructions to digital education. For economic outcomes, there was very limited evidence.

Overall, most interventions analysed had generally ineffective or mixed-effect outcomes. In the case of multifaceted strategies, it was not possible to define the contribution of each one and their specific attributes in the results, or to identify the synergistic effect of the interventions [68]. Our results were similar to those observed in the study by Grimshaw et al., in which the majority of evaluated strategies showed modest to moderate improvements in care. Grimshaw's systematic review was the most comprehensive identified, without restriction as to the type of strategy or clinical area. In that review, 235 studies were evaluated, with most having evaluated process measures as the primary outcome. The isolated interventions that were most commonly evaluated were reminders, dissemination of educational materials, and auditing and feedback. The authors concluded that there was an insufficient evidence base to point to strategies with the greatest potential to be effective in different contexts of guideline implementation [58].

In general, educational strategies have been widely addressed in the literature across a large number of studies, and regardless of whether they are the most effective strategy, they have presented important information to be targeted to specific groups [25, 52, 55, 63]. The small number of comparisons between educational interventions with more complex strategies involving large-scale changes and higher cost [55] results in evidence gaps, and in a tendency to value educational approaches that require fewer resources and are easier to adopt by guideline developers or implementers with limited funding [69], possibly obtaining moderate results that are unlikely to be contradicted by other study designs.

Results for educational meetings similar to ours were reported in a recent systematic review, in which it was observed that this strategy promoted modest improvement in professional practice and, to a lesser degree, in patient outcomes. Educational meetings can improve compliance with desired practice, and the results of using this strategy can be leveraged when used in conjunction with other approaches [70]. This result is corroborated by previous studies, where multifaceted educational interventions for knowledge translation seem to be more effective in improving professional practice outcomes [51], but not necessarily in improving treatment outcomes for patients [71, 72]. However, the heterogeneity of interventions described as educational strategies, presenting different teaching and learning methods, makes it difficult to conduct a more detailed comparison between each of the proposed interventions [52].

Reminders have also been considered low-cost and low-complexity approaches. Results in the literature have been modest but indicated that reminders can be effective in changing the behaviour of professionals [33, 73]. The use of reminders designed for specific needs may be more likely to succeed, and reminders that prompted or required professionals’ responses were more likely to be effective in changing behaviour [33]. In our overview, we did not indicate which features of the reminder systems could promote better results [73], but a simpler format, such as manual reminders delivered on paper, can show low and moderate results in behaviour change, and can be used as a single intervention to improve quality of service [74]. Literature on the use of electronic reminders applied to health professionals, such as pharmacists, to support practice change have presented controversial results, but studies with a more robust methodology may indicate greater efficacy in the community pharmacy setting [55].

Audit and feedback may be a relevant strategy to identify the coherence between the recommendation and what is practised by the healthcare providers. In an overview of systematic reviews, this strategy was generally effective in improving both the care process and clinical outcomes, although the authors did not consider the statistical significance of the results [32]. Providing continuous feedback to professionals is an important strategy to increase professionals’ awareness of the impact of their practice and manager support for decision-making [26]. An important literature review indicated that audit and feedback may be responsible for a small, but potentially important, benefit for professional practice, varying based on the way the intervention is designed and delivered. According to the analyses, feedback may be more effective when provided by a supervisor or senior colleague, delivered at least monthly, both verbally and in written format, and when it includes explicit targets and an action plan [75].

Two interventions that were relatively rarely addressed in the included systematic reviews, but with promising results, were care pathway and organizational culture. Care pathway is an intervention that involves the standardization of care processes and its implementation is usually complex, being more frequently used for diseases and high-cost situations [76]. In the case of our results, most of them came from studies in the cardiovascular area, which could support more comprehensive activities to implement guidelines in this clinical area. Organizational culture is also a more complex and costly intervention targeted at healthcare organizations. These interventions can be implemented by promoting, for example, revisions of local procedures, protocols and tasks [77].

Behaviour change of the team is another important factor to consider in the guideline implementation process. A pioneering study using psychological theory to identify barriers to implementation of clinical guidelines and evidence-based practice identified 12 different domains of behaviour change [78]. Therefore, when the literature review reveals many studies focusing on educational strategies—that is, only on the education domain—there is a lack of more complex studies to understand professional and organizational behaviour change, which could help to determine what strategies would be more effective in different circumstances [57]. Moreover, leadership presence and incentive policies [40], or even interventions targeting the entire multidisciplinary team, seem to be more commonly accepted in the strategies for guideline implementation and dissemination [60].

Once awareness of the critical points that can compromise the implementation of a clinical guideline has been established, targeted strategies can be used to overcome barriers. A literature review reported that interventions tailored to prospectively identified barriers are more likely to improve professional practice than no intervention or guideline dissemination. However, methods to identify barriers and adapt interventions to address these barriers need further improvement, and further research is needed to assess the effectiveness of tailored interventions in comparison with other interventions [79].

Adherence of both professionals and organizations to guidelines can be improved when they are developed locally or adapted to the local context, taking into account issues such as value judgements, use of resources, characteristics of the local context and feasibility [26]. In the implementation of very specific guidelines, analysis of local context may be even more relevant, and it can make a difference in, for example, prescription of medications (involving normative and structural issues), or conduct of specific services such as intensive care units [39, 49].

In view of the substantial heterogeneity among interventions and the wide range of areas and follow-ups to be studied, perhaps more important than a standard study is further research on a systematic analysis of context and a theoretical framework of implementation. Studies should explore the features of an intervention that are effective in a specific context and how this could be translated into another context [42]. It is worth mentioning that, in general, tailored implementation interventions should not be considered transferable between different conditions or countries [80].

A recent study described the process and results obtained with a project developed to identify barriers to the national childbirth guidelines in Brazil and strategies for implementation. After identifying and prioritizing barriers to implementation, a deliberative dialogue was undertaken to discuss options for addressing them based on an evidence synthesis. As a result, the following interventions were selected: promoting the use of multifaceted interventions, educational interventions, audit and feedback to adjust professional practice, and reminders to mediate the interaction between workers and service users; enabling patient-mediated interventions; and engaging opinion leaders to promote the use of guidelines [81]. In initiatives like this, the present study has the potential to provide an evidence map organized by intervention target, intended outcome and results achieved.

### Strengths and limitations

The results presented in this overview were based on secondary data, and where necessary primary data was collected. Therefore, the first limitation is related to the lack of detailed information on the strategies and outcomes reported by the authors of the primary studies. Moreover, with regard to multifaceted interventions, some systematic reviews presented the main strategy without listing the other strategies used in combination with the main one.

Second, we used the EPOC taxonomy to classify the implementation interventions, but some systematic reviews, especially those prior to EPOC classification, had used their own categorization. In order to standardize the classification according to EPOC, we categorized some strategies based on data from the systematic reviews. In some cases, such reclassification may not entirely reflect the intervention addressed in the primary study, so this may have caused the results to appear more or less effective for each strategy.

Third, the wide scope and difficulty in gathering a large amount of information from different contexts in a comprehensible way should be taken into consideration, and the analysis of the results should consider this diversity (e.g. the level of development of the countries, types of services where strategies were implemented, clinical areas, attributes of each intervention). It should be mentioned that it was not our intention to conduct a meta-analysis of effectiveness data, but to present the strategies with a large number of analyses and a statistically significant impact on any of the outcomes evaluated.

The fourth limitation relates to the way that the results were tabulated to categorize the effectiveness of the strategies. The focus of the analysis was on positive results with statistical significance. However, many studies that assess guideline dissemination and implementation strategies are cluster-randomized controlled trials, which present unit-of-analysis errors that make it difficult to make precise estimates regarding the statistical significance of the strategies [82].

## Conclusion

Generally, national clinical guideline developers are not responsible for implementation and may leave it to regional or local groups. However, guideline implementation may require a national approach that provides a basis for effective use at the local level. The data presented in this overview can serve as an important source of information, while more robust evidence may establish a coherent relationship between professional and organizational behaviour to better inform the choice of interventions, and to evaluate the efficiency of dissemination and implementation strategies in the presence of different barriers and facilitators.

Further research is needed to compare more complex implementation strategies, as simple strategies reported with good results in the literature can be used in early interventions. The decision-making of managers should be based on the whole context of the health service, the evidence available so far, and the best use of resources. Sometimes the implementation of a guideline can be justified in a specific field or area, but it is important to take scarce resources into consideration when prioritizing actions and strategies that may contribute to improve practices in health services.

Therefore, the identification and assessment of the main factors related to the guideline implementation process and the discussion of the strategies addressed in this overview are relevant in facilitating the direction and decision-making of guideline implementers. Even if the included literature is unanimous in highlighting the various limitations related to the lack of standardization, low methodological quality of the studies, and especially the lack of conclusions about the superiority of one strategy over another [26, 54, 58], the summary of the results of this overview provides information on the strategies that have been most widely studied in the last few years and their effectiveness in the context in which they were applied. The identification of barriers, facilitators, perspectives of behaviour change and context, combined with the results from the best available evidence, can be an important tool for guideline implementation.

Thus, this panorama can support strategy decision-making adequate for the SUS and other health systems, seeking to positively impact on the appropriate use of guidelines, healthcare outcomes and the sustainability of the SUS.

## Supplementary Information

**Additional file 1.** Literature search.

**Additional file 2.** Excluded studies.

**Additional file 3.** Characteristics and AMSTAR2 of the systematic reviews.

**Additional file 4.** Effectiveness of guideline implementation strategies from systematic reviews by type of outcome.

## Data Availability

The datasets used and/or analysed during the current study are available from the corresponding author on reasonable request.
