# Supplementary material for: Strategies for the implementation of clinical practice guidelines in public health: an overview of systematic reviews
Source: Health Res Policy Syst. 2022 Jan 24;20:13. doi: 10.1186/s12961-022-00815-4 (PMC8785489; doi:10.1186/s12961-022-00815-4)
Supplement: Supplementary file 1 — Additional file 1. Literature search. [file 12961_2022_815_MOESM1_ESM.pdf]

Additional file 1 - Literature search

	**Search until June 19, 2017**	
	**PUBMED (NCBI)**	
#1	(((("Practice Guidelines as Topic"[Mesh]) OR "Practice Guideline" [Publication Type]) OR Clinical Protocols[MeSH Terms]) OR Critical Pathways[MeSH Terms]) OR Health Planning Guidelines[MeSH Terms]	271812
#2	(((((((((((((((((("Guideline Adherence"[Mesh]) OR "Health Plan Implementation"[Mesh]) OR implement*) OR aware*) OR uptake) OR up-take) OR "take up") OR take-up) OR adhere) OR adhered) OR adherence) OR concordance) OR accordance) OR adopt*) OR comply) OR complies) OR compliance) OR disseminat*) OR spread) OR spreading) OR barrier*) OR facilitat*	2140617
#3	((((("Meta-Analysis" [Publication Type]) OR "Meta-Analysis as Topic"[Mesh]) OR meta-analysis[Title/Abstract]) OR ((systematic[Title/Abstract]) AND review[Title/Abstract]))) NOT ((((((case[Title]) AND report[Title])) OR "Editorial" [Publication Type]) OR "Comment" [Publication Type]) OR "Newspaper Article" [Publication Type]) OR "Letter" [Publication Type])	194567
#4	#1 AND #2 AND #3	1581
	**CRD**	
#1	((MeSH DESCRIPTOR Practice Guidelines as Topic EXPLODE ALL TREES) OR (MeSH DESCRIPTOR Clinical Protocols EXPLODE ALL TREES) OR (MeSH DESCRIPTOR Health Planning Guidelines EXPLODE ALL TREES) OR (MeSH DESCRIPTOR Critical Pathways EXPLODE ALL TREES))	2224
#2	((MeSH DESCRIPTOR Guideline Adherence EXPLODE ALL TREES) OR (MeSH DESCRIPTOR Health Plan Implementation EXPLODE ALL TREES) OR (implement*) OR (aware*) OR (uptake) OR (up-take) OR (take up) OR (take-up) OR (adhere) OR (adhered) OR (adherence) OR (concordance) OR (accordance) OR (adopt*) OR (comply) OR (compliance) OR (complies) OR (disseminat*) OR (spread) OR (spreading) OR (barrier*) OR (facilitat*))	13890
#3	(#1) AND (#1) IN DARE, HTA	185
	**SCOPUS**	
#1	(TITLE-ABS-KEY ("Practice Guideline") OR TITLE-ABS-KEY ("Practice Guidelines") OR TITLE-ABS-KEY ("Clinical Protocol") OR TITLE-ABS-KEY ("Clinical Protocols") OR TITLE-ABS-KEY ("Health Planning Guideline") OR TITLE-ABS-KEY ("Health Planning Guidelines") OR TITLE-ABS-KEY ("Critical Pathway") OR TITLE-ABS-KEY ("Critical Pathways"))	403953
#2	(TITLE-ABS-KEY (implementation) OR TITLE-ABS-KEY (adherence))	1164698
#3	(TITLE-ABS-KEY ("meta-analysis") OR TITLE-ABS-KEY (meta AND analysis) OR TITLE-ABS-KEY (systematic AND review))	353340
#4	#1 AND #2 AND #3	2338 (1297+1041)

#1	'practice guideline':ab,ti OR 'clinical pathway':ab,ti OR 'clinical protocol':ab,ti OR 'health care planning':ab,ti	10610
#2	'protocol compliance':ab,ti OR 'implementation':ab,ti OR 'aware':ab,ti OR 'uptake':ab,ti OR 'up-take':ab,ti OR 'take up':ab,ti OR 'take-up':ab,ti OR 'spread':ab,ti OR 'spreading':ab,ti OR 'disseminat*':ab,ti OR 'adhere':ab,ti OR 'adhered':ab,ti OR 'adherence':ab,ti OR 'barrie*':ab,ti OR 'facilitat*':ab,ti OR 'concordance':ab,ti OR 'accordance':ab,ti OR 'adopt*':ab,ti OR 'comply':ab,ti OR 'complies':ab,ti OR 'compliance':ab,ti	2232887
#3	#1 AND #2	3090
#4	#3 NOT 'medline'	1332
	**Cochrane**	
#1	"Practice Guideline" OR "Practice Guidelines" in Title, Abstract, Keywords or "Clinical Protocols" OR "Clinical Protocol" in Title, Abstract, Keywords or "Critical Pathways" OR "Critical Pathway" in Title, Abstract, Keywords or "Health Planning Guidelines" OR "Health Planning Guideline" in Title, Abstract, Keywords in Cochrane Reviews'	Cochrane Reviews (69)<br>Other Reviews (451)
#2	'"Guideline Adherence" OR "Health Plan Implementation" OR implement* or aware* OR uptake OR up-take OR “take up” OR take-up OR adhere OR adhered OR adherence OR concordance OR accordance OR adopt* OR comply or complies or compliance or disseminat* OR spread OR spreading OR barrier or barriers OR facilitat* in Title, Abstract, Keywords in Cochrane Reviews'	Cochrane Reviews (2290)<br>Other Reviews (928)
#3	#1 AND #2	76
#4	MeSH descriptor: [Practice Guideline] explode all trees	16
#5	MeSH descriptor: [Practice Guidelines as Topic] explode all trees	2081
		Cochrane Reviews (81)<br>Other Reviews (968)
	**Web of Science**	
#1	Tópico: ("Practice Guidelines") OR Tópico: ("Clinical Protocols") OR Tópico: ("Critical Pathways") OR Tópico: ("Health Planning Guidelines")	28694
#2	Tópico: ("Guideline Adherence") OR Tópico: ("Health Plan Implementation") OR Tópico: (implement*) OR Tópico: (aware*) OR Tópico: (uptake) OR Tópico: (up-take) OR Tópico: ("take up") OR Tópico: (take-up) OR Tópico: (adhere) OR Tópico: (adhered) OR Tópico: (adherence) OR Tópico: (concordance) OR Tópico: (accordance) OR Tópico: (adopt*) OR Tópico: (comply) OR Tópico: (complies) OR Tópico: (compliance) OR Tópico: (disseminat*) OR Tópico: (spread) OR Tópico: (spreading) OR Tópico: (barrier*) OR Tópico: (facilitat*)	4136252
#3	Tópico: ("meta-analysis") OR Tópico: ("meta analysis") OR Tópico: ("systematic review")	175883

#4	#1 AND #2 AND #3	436
	**Rx for change**	
#1	Guideline implementation	31
#2	Guideline Adherence	106
#3	Clinical Protocols	281
#4	Practice Guidelines	125
	TOTAL	543
	**Health Systems Evidence**	
#1	("Practice Guidelines" OR "Clinical Protocols" OR "Critical Pathways" OR "Health Planning Guidelines") AND (adherence or implementation)	103
	**Epistemonikos**	
#1	title:((Practice Guideline*) OR (Clinical Protocol*) OR (Critical Pathway*) OR (Health Planning Guideline*)) AND title:((Guideline Adherence) OR (Health Plan Implementation) OR implement* OR aware* OR uptake OR up-take OR "take up" OR take-up OR adhere OR adhered OR adherence OR concordance OR accordance OR adopt* OR comply OR complies OR compliance OR disseminat* OR spread OR spreading OR barrier* OR facilitat*) AND (title:("meta-analysis" OR "meta analysis" OR "systematic review") OR abstract:("meta-analysis" OR "meta analysis" OR "systematic review"))	14
	**Cinahl**	
#1	MW practice guidelines as topic OR MW clinical protocols OR MW critical pathways OR MW Health Planning Guidelines OR AB practice guidelines	4277
#2	MW guideline adherence OR MW Health Plan Implementation OR AB (implement* or aware* or uptake or up-take or take-up or adhere or adhered or adherence or concordance or accordance or adopt* or comply or complies or compliance or disseminat* or spread or spreading or barrier* or facilitat*) OR TI (implement* or aware* or uptake or up-take or take-up or adhere or adhered or adherence or concordance or accordance or adopt* or comply or complies or compliance or disseminat* or spread or spreading or barrier* or facilitat*)	158791
#3	*#1 AND #2 Limitadores*<br>**Remove:**Texto completo<br>**Remove:**Excluir registros do MEDLINE	440

Search update from 19 June 2017 to 10 August 2019		
**PUBMED (NCBI)**		
#1	(((("Practice Guidelines as Topic"[Mesh]) OR "Practice Guideline" [Publication Type]) OR Clinical Protocols[MeSH Terms]) OR Critical Pathways[MeSH Terms]) OR Health Planning Guidelines[MeSH Terms]	19493
#2	((((((((((((((((("Guideline Adherence"[Mesh]) OR "Health Plan Implementation"[Mesh]) OR implement*) OR aware*) OR uptake) OR up-take) OR “take up") OR take-up) OR adhere) OR adhered) OR adherence) OR concordance) OR accordance) OR adopt*) OR comply) OR complies) OR compliance) OR disseminat*) OR spread) OR spreading) OR barrier*) OR facilitat*	368083
#3	((((("Meta-Analysis" [Publication Type]) OR "Meta-Analysis as Topic"[Mesh]) OR meta-analysis[Title/Abstract]) OR ((systematic[Title/Abstract]) AND review[Title/Abstract]))) NOT ((((((case[Title]) AND report[Title])) OR "Editorial" [Publication Type]) OR "Comment" [Publication Type]) OR "Newspaper Article" [Publication Type]) OR "Letter" [Publication Type])	68326
#4	#1 AND #2 AND #3	**322**

CRD		
#1	((MeSH DESCRIPTOR Practice Guidelines as Topic EXPLODE ALL TREES) OR (MeSH DESCRIPTOR Clinical Protocols EXPLODE ALL TREES) OR (MeSH DESCRIPTOR Health Planning Guidelines EXPLODE ALL TREES) OR (MeSH DESCRIPTOR Critical Pathways EXPLODE ALL TREES))	3
#2	((MeSH DESCRIPTOR Guideline Adherence EXPLODE ALL TREES) OR (MeSH DESCRIPTOR Health Plan Implementation EXPLODE ALL TREES) OR (implement*) OR (aware*) OR (uptake) OR (up-take) OR (take up) OR (take-up) OR (adhere) OR (adhered) OR (adherence) OR (concordance) OR (accordance) OR (adopt*) OR (comply) OR (compliance) OR (complies) OR (disseminat*) OR (spread) OR (spreading) OR (barrier*) OR (facilitat*))	63
#3	(#1) AND (#2) IN DARE, HTA	**1**

SCOPUS		
#1	(TITLE-ABS-KEY ("Practice Guideline") OR TITLE-ABS-KEY ("Practice Guidelines") OR TITLE-ABS-KEY ("Clinical Protocol") OR TITLE-ABS-KEY ("Clinical Protocols") OR TITLE-ABS-KEY ("Health Planning Guideline") OR TITLE-ABS-KEY ("Health Planning Guidelines") OR TITLE-ABS-KEY ("Critical Pathway") OR TITLE-ABS-KEY ("Critical Pathways"))	71740
#2	(TITLE-ABS-KEY (implementation) OR TITLE-ABS-KEY (adherence))	256465
#3	(TITLE-ABS-KEY ("meta-analysis") OR TITLE-ABS-KEY (meta AND analysis) OR TITLE-ABS-KEY (systematic AND review))	128591
#4	#1 AND #2 AND #3	**916**

Embase		
#1	'practice guideline':ab,ti OR 'clinical pathway':ab,ti OR 'clinical protocol':ab,ti OR 'health care planning':ab,ti	10610
#2	'protocol compliance':ab,ti OR 'implementation':ab,ti OR 'aware':ab,ti OR 'uptake':ab,ti OR 'up-take':ab,ti OR 'take up':ab,ti OR 'take-up':ab,ti OR 'spread':ab,ti OR 'spreading':ab,ti OR 'disseminat*':ab,ti OR 'adhere':ab,ti OR 'adhered':ab,ti OR 'adherence':ab,ti OR 'barrie*':ab,ti OR 'facilitat*':ab,ti OR	2676110

	'concordance':ab,ti OR 'accordance':ab,ti OR 'adopt*':ab,ti OR 'comply':ab,ti OR 'complies':ab,ti OR 'compliance':ab,ti	
#3	#1 AND #2	3999
#4	#3 NOT 'medline' AND [1-7-2017]/sd NOT [31-7-2019]/sd	**440**
	**Cochrane**	
#1	"Practice Guideline" OR "Practice Guidelines" in Title, Abstract, Keywords or "Clinical Protocols" OR "Clinical Protocol" in Title, Abstract, Keywords or "Critical Pathways" OR "Critical Pathway" in Title, Abstract, Keywords or "Health Planning Guidelines" OR "Health Planning Guideline" in Title, Abstract, Keywords in Cochrane Reviews'	Cochrane Reviews (14) Other Reviews (0)
#2	'"Guideline Adherence" OR "Health Plan Implementation" OR implement* or aware* OR uptake OR up-take OR "take up" OR take-up OR adhere OR adhered OR adherence OR concordance OR accordance OR adopt* OR comply or complies or compliance or disseminat* OR spread OR spreading OR barrier or barriers OR facilitat* in Title, Abstract, Keywords in Cochrane Reviews'	Cochrane Reviews (380) Other Reviews (0)
#3	#1 AND #2	**9**
	MeSH descriptor: [Practice Guideline] explode all trees	0
	MeSH descriptor: [Practice Guidelines as Topic] explode all trees	0
	**Web of Science**	
#1	Tópico: ("Practice Guidelines") OR Tópico: ("Clinical Protocols") OR Tópico: ("Critical Pathways") OR Tópico: ("Health Planning Guidelines")	8413
#2	Tópico: ("Guideline Adherence") OR Tópico: ("Health Plan Implementation") OR Tópico: (implement*) OR Tópico: (aware*) OR Tópico: (uptake) OR Tópico: (up-take) OR Tópico: ("take up") OR Tópico: (take-up) OR Tópico: (adhere) OR Tópico: (adhered) OR Tópico: (adherence) OR Tópico: (concordance) OR Tópico: (accordance) OR Tópico: (adopt*) OR Tópico: (comply) OR Tópico: (complies) OR Tópico: (compliance) OR Tópico: (disseminat*) OR Tópico: (spread) OR Tópico: (spreading) OR Tópico: (barrier*) OR Tópico: (facilitat*)	1030981
#3	Tópico: ("meta-analysis") OR Tópico: ("meta analysis") OR Tópico: ("systematic review")	83993
#4	#1 AND #2 AND #3	**237**
	**Rx for change**	
#1	Guideline implementation	0
#2	Guideline Adherence	0
#3	Clinical Protocols	0
#4	Practice Guidelines	0
	TOTAL	0
	**Health Systems Evidence**	
#1	("Practice Guidelines" OR "Clinical Protocols" OR "Critical Pathways" OR "Health Planning Guidelines") AND (adherence or implementation)	**6**
	**Epistemonikos**	
	title:((Practice Guideline*) OR (Clinical Protocol*) OR (Critical Pathway*) OR (Health Planning Guideline*)) AND title:((Guideline Adherence) OR (Health Plan Implementation) OR implement* OR aware* OR uptake OR up-take OR "take up" OR take-up OR adhere OR adhered OR adherence OR concordance OR accordance OR adopt* OR comply OR complies OR compliance OR disseminat* OR spread OR spreading OR barrier* OR facilitat*) AND (title:("meta-analysis" OR "meta analysis" OR "systematic review") OR abstract:("meta-analysis" OR "meta analysis" OR "systematic review"))	**12**

Cinahl		
#1	MW practice guidelines as topic OR MW clinical protocols OR MW critical pathways OR MW Health Planning Guidelines OR AB practice guidelines	1415
#2	MW guideline adherence OR MW Health Plan Implementation OR AB (implement* or aware* or uptake or up-take or take-up or adhere or adhered or adherence or concordance or accordance or adopt* or comply or complies or compliance or disseminat* or spread or spreading or barrier* or facilitat*) OR TI (implement* or aware* or uptake or up-take or take-up or adhere or adhered or adherence or concordance or accordance or adopt* or comply or complies or compliance or disseminat* or spread or spreading or barrier* or facilitat*)	57442
#3	*#1 AND #2 Limitadores*<br>**Remove:**Texto completo<br>**Remove:**Excluir registros do MEDLINE	**17**
