# Supplementary material for: Strategies for the implementation of clinical practice guidelines in public health: an overview of systematic reviews
Source: Health Res Policy Syst. 2022 Jan 24;20:13. doi: 10.1186/s12961-022-00815-4 (PMC8785489; doi:10.1186/s12961-022-00815-4)
Supplement: Supplementary file 2 — Additional file 2. Excluded studies. [file 12961_2022_815_MOESM2_ESM.docx]

| **Additional file 2 – Excluded studies** | |
| --- | --- |
| **Reference** | **Reason for exclusion** |
| Ackermann SP, Cheal N. Factors affecting physician adherence to breast cancer screening guidelines. J Cancer Educ. 1994 Summer;9(2):96-100. doi: 10.1080/08858199409528279. PMID: 7917899. | Outcome |
| Addington D, Kyle T, Desai S, Wang J. Facilitators and barriers to implementing quality measurement in primary mental health care: Systematic review. Can Fam Physician. 2010 Dec;56(12):1322-31. PMID: 21375065; PMCID: PMC3001932. | Outcome |
| Ahmad, A.; Sorensen, K. Enabling and hindering factors influencing adherence to asthma treatment among adolescents: A systematic literature review, Journal of Asthma, 53:8, 862-878, 2016. DOI: 10.3109/02770903.2016.1155217 | Outcome |
| Al-Ansary LA, Tricco AC, Adi Y, Bawazeer G, Perrier L, Al-Ghonaim M, AlYousefi N, Tashkandi M, Straus SE. A systematic review of recent clinical practice guidelines on the diagnosis, assessment and management of hypertension. PLoS One. 2013;8(1):e53744. doi: 10.1371/journal.pone.0053744. Epub 2013 Jan 17. PMID: 23349738; PMCID: PMC3547930. | Outcome |
| Aminzadeh F. Adherence to recommendations of community-based comprehensive geriatric assessment programmes. Age Ageing. 2000 Sep;29(5):401-7. doi: 10.1093/ageing/29.5.401. PMID: 11108411. | Outcome |
| Amorin-Woods LG, Beck RW, Parkin-Smith GF, Lougheed J, Bremner AP. Adherence to clinical practice guidelines among three primary contact professions: a best evidence synthesis of the literature for the management of acute and subacute low back pain. J Can Chiropr Assoc. 2014 Sep;58(3):220-37. PMID: 25202150; PMCID: PMC4139767. | Outcome |
| Andreev, P.; Michalowski, W.; Kuziemsky, C.; Hadjiyannakis, S. ActCPG framework to learn about information user requirements of a clinical practice guideline. Health Policy and Technology 2012;1(3):165-172. https://doi.org/10.1016/j.hlpt.2012.07.005 | Study design |
| Armstrong MJ, Rueda JD, Gronseth GS, Mullins CD. Framework for enhancing clinical practice guidelines through continuous patient engagement. Health Expect. 2017 Feb;20(1):3-10. doi: 10.1111/hex.12467. Epub 2016 Apr 26. PMID: 27115476; PMCID: PMC5217879. | Study design |
| Asonganyi E, Vaghasia M, Rodrigues C, Phadtare A, Ford A, Pietrobon R, Atashili J, Lynch C. Factors affecting compliance with clinical practice guidelines for pap smear screening among healthcare providers in africa: systematic review and meta-summary of 2045 individuals. PLoS One. 2013 Sep 12;8(9):e72712. doi: 10.1371/journal.pone.0072712. PMID: 24069156; PMCID: PMC3771969. | Outcome |
| Augustin M, Holland B, Dartsch D, Langenbruch A, Radtke MA. Adherence in the treatment of psoriasis: a systematic review. Dermatology. 2011;222(4):363-74. doi: 10.1159/000329026. Epub 2011 Jul 13. PMID: 21757881. | Outcome |
| Baatiema L, Otim ME, Mnatzaganian G, de-Graft Aikins A, Coombes J, Somerset S. Health professionals' views on the barriers and enablers to evidence-based practice for acute stroke care: a systematic review. Implement Sci. 2017 Jun 5;12(1):74. doi: 10.1186/s13012-017-0599-3. PMID: 28583164; PMCID: PMC5460544. | Outcome |
| Barbieri A, Milan E, Cattaneo MG, Faggian F, Panella M. I percorsi assistenziali migliorano gli outcome dei pazienti affetti da scompenso cardiaco? [Do critical pathways improve outcomes of patients with cardiac failure?]. Ig Sanita Pubbl. 2011 Sep-Oct;67(5):591-606. Italian. PMID: 22508610. | Outcome |
| Bayona H, Owolabi M, Feng W, Olowoyo P, Yaria J, Akinyemi R, Sawers JR, Ovbiagele B. A systematic comparison of key features of ischemic stroke prevention guidelines in low- and middle-income vs. high-income countries. J Neurol Sci. 2017 Apr 15;375:360-366. doi: 10.1016/j.jns.2017.02.040. Epub 2017 Feb 20. PMID: 28320168; PMCID: PMC5813247. | Outcome |
| Bekkering GE, Engers AJ, Wensing M, Hendriks HJ, van Tulder MW, Oostendorp RA, Bouter LM. Development of an implementation strategy for physiotherapy guidelines on low back pain. Aust J Physiother. 2003;49(3):208-14. doi: 10.1016/s0004-9514(14)60240-3. PMID: 12952520. | Study design |
| Bernardy, N. C.; Hamblen, J. L.; Friedman, M. J.; Kivlahan, D. R. Co-occurring Posttraumatic Stress Disorder and Substance Use Disorder: Recommendations for Management and Implementation in the Department of Veterans Affairs, Journal of Dual Diagnosis, 7:4, 242-261, 2011. DOI: 10.1080/15504263.2011.620446 | Study design |
| Berra K. Does nurse case management improve implementation of guidelines for cardiovascular disease risk reduction? J Cardiovasc Nurs. 2011 Mar-Apr;26(2):145-67. doi: 10.1097/JCN.0b013e3181ec1337. PMID: 21076315. | Outcome |
| Bérubé, M.-È., Poitras, S., Bastien, M., Laliberté, L.-A., Lacharité, A., & Gross, D. P. Strategies to translate knowledge related to common musculoskeletal conditions into physiotherapy practice: a systematic review. Physiotherapy, 2018; 104(1): 1–8. doi:10.1016/j.physio.2017.05.002 | Outcome |
| Bonetti D, Eccles M, Johnston M, Steen N, Grimshaw J, Baker R, Walker A, Pitts N. Guiding the design and selection of interventions to influence the implementation of evidence-based practice: an experimental simulation of a complex intervention trial. Soc Sci Med. 2005 May;60(9):2135-47. doi: 10.1016/j.socscimed.2004.08.072. Epub 2004 Dec 8. PMID: 15743661. | Study design |
| Brooks SC, Morrison LJ. Implementation of therapeutic hypothermia guidelines for post-cardiac arrest syndrome at a glacial pace: seeking guidance from the knowledge translation literature. Resuscitation. 2008 Jun;77(3):286-92. doi: 10.1016/j.resuscitation.2008.01.017. Epub 2008 Mar 7. PMID: 18329157. | Study design |
| Brosseau L, Rahman P, Toupin-April K, Poitras S, King J, De Angelis G, Loew L, Casimiro L, Paterson G, McEwan J. A systematic critical appraisal for non-pharmacological management of osteoarthritis using the appraisal of guidelines research and evaluation II instrument. PLoS One. 2014 Jan 10;9(1):e82986. doi: 10.1371/journal.pone.0082986. PMID: 24427268; PMCID: PMC3888378. | Outcome |
| Brouwers, M.C., Garcia, K., Makarski, J. et al. The landscape of knowledge translation interventions in cancer control: What do we know and where to next? A review of systematic reviews. Implementation Sci 6, 130 (2011). https://doi.org/10.1186/1748-5908-6-130 | Study design |
| Byers T. Implementation of the new American Cancer Society process for creating cancer screening guidelines. Cancer Cytopathol. 2012 Oct 25;120(5):287-90. doi: 10.1002/cncy.21198. Epub 2012 Apr 19. PMID: 22517634. | Study design |
| Cabrera, P. A., & Pardo, R. Review of evidence based clinical practice guidelines developed in Latin America and Caribbean during the last decade: an analysis of the methods for grading quality of evidence and topic prioritization. Globalization and Health, 2019; 15(1). doi:10.1186/s12992-019-0455-0 | Outcome |
| Cahill NE, Heyland DK. Bridging the guideline-practice gap in critical care nutrition: a review of guideline implementation studies. JPEN J Parenter Enteral Nutr. 2010 Nov-Dec;34(6):653-9. doi: 10.1177/0148607110361907. PMID: 21097765. | Study design |
| Chan, W. V., Pearson, T. A., Bennett, G. C., Cushman, W. C., Gaziano, T. A., Gorman, P. N., … Wells, B. L. ACC/AHA Special Report: Clinical Practice Guideline Implementation Strategies: A Summary of Systematic Reviews by the NHLBI Implementation Science Work Group. Journal of the American College of Cardiology, 2017; 69(8): 1076–1092. doi:10.1016/j.jacc.2016.11.004 | Study design |
| Chau J, Thompson DR, Chan D, Chung L, Au WL, Tam S, Fung G, Lo S, Chow V. An evaluation of the implementation of a best practice guideline on tracheal suctioning in intensive care units. Int J Evid Based Healthc. 2007 Sep;5(3):354-9. doi: 10.1111/j.1479-6988.2007.00073.x. PMID: 21631796. | Study design |
| Clarke A, Blundell N, Forde I, Musila N, Spitzer D, Naqvi S, Browne J. Can guidelines improve referral to elective surgical specialties for adults? A systematic review. Qual Saf Health Care. 2010 Jun;19(3):187-94. doi: 10.1136/qshc.2008.029918. Epub 2010 Mar 8. PMID: 20211956; PMCID: PMC2989157. | Outcome |
| Conrardy JA, Brenek B, Myers S. Determining the state of knowledge for implementing the universal protocol recommendations: an integrative review of the literature. AORN J. 2010 Aug;92(2):194-207. doi: 10.1016/j.aorn.2009.12.031. PMID: 20678608. | Outcome |
| Cornell E, Chandhok L, Rubin K. Implementation of referral guidelines at the interface between pediatric primary and subspecialty care. Healthc (Amst). 2015 Jun;3(2):74-9. doi: 10.1016/j.hjdsi.2015.02.003. Epub 2015 Feb 27. PMID: 26179727. | Intervention |
| Davis DA, Taylor-Vaisey A. Translating guidelines into practice. A systematic review of theoretic concepts, practical experience and research evidence in the adoption of clinical practice guidelines. CMAJ. 1997 Aug 15;157(4):408-16. PMID: 9275952; PMCID: PMC1227916. | Outcome |
| Doumit, D.; Giehl, J.Evaluation of Internal Quality Management in Hospitals and Ambulatory Health Care - An Analysis of the Status of Implementation and of Efficacy/Effectiveness and Benefit. Gesundheitsokonomie und Qualitatsmanagement 2015;20(6):278-285 | Outcome |
| Dulko D, Hertz E, Julien J, Beck S, Mooney K. Implementation of cancer pain guidelines by acute care nurse practitioners using an audit and feedback strategy. J Am Acad Nurse Pract. 2010 Jan;22(1):45-55. doi: 10.1111/j.1745-7599.2009.00469.x. PMID: 20074196. | Outcome |
| Dunkley AJ, Bodicoat DH, Greaves CJ, Russell C, Yates T, Davies MJ, Khunti K. Diabetes prevention in the real world: effectiveness of pragmatic lifestyle interventions for the prevention of type 2 diabetes and of the impact of adherence to guideline recommendations: a systematic review and meta-analysis. Diabetes Care. 2014 Apr;37(4):922-33. doi: 10.2337/dc13-2195. Erratum in: Diabetes Care. 2014 Jun;37(6):1775-6. PMID: 24652723. | Study design |
| Durieux P, Ravaud P, Dosquet P, Durocher A. Mise en oeuvre des recommandations médicales : revue systématique des revues systématiques [Effectiveness of clinical guideline implementation strategies: systematic review of systematic reviews]. Gastroenterol Clin Biol. 2000 Nov;24(11):1018-25. French. PMID: 11139669. | Study design |
| Ebrahim S. Detection, adherence and control of hypertension for the prevention of stroke: a systematic review. Health Technol Assess. 1998;2(11):i-iv, 1-78. PMID: 9789758. | Outcome |
| Egerton T, Diamond LE, Buchbinder R, Bennell KL, Slade SC. A systematic review and evidence synthesis of qualitative studies to identify primary care clinicians' barriers and enablers to the management of osteoarthritis. Osteoarthritis Cartilage. 2017 May;25(5):625-638. doi: 10.1016/j.joca.2016.12.002. Epub 2016 Dec 7. PMID: 27939622. | Outcome |
| El Baz N, Middel B, van Dijk JP, Oosterhof A, Boonstra PW, Reijneveld SA. Are the outcomes of clinical pathways evidence-based? A critical appraisal of clinical pathway evaluation research. J Eval Clin Pract. 2007 Dec;13(6):920-9. doi: 10.1111/j.1365-2753.2006.00774.x. PMID: 18070263. | Outcome |
| Engel J, Damen NL, van der Wulp I, de Bruijne MC, Wagner C. Adherence to Cardiac Practice Guidelines in the Management of Non-ST-Elevation Acute Coronary Syndromes: A Systematic Literature Review. Curr Cardiol Rev. 2017;13(1):3-27. doi: 10.2174/1573403x12666160504100025. PMID: 27142050; PMCID: PMC5324326. | Outcome |
| Etxeberría Aguirre, A.; Rotaeche Del Campo, R. Evidence-based clinical practice guidelines: Current development and future perspectives. Revista de Calidad Asistencial 2006;21(5):228-237 | Study design |
| Farquhar CM, Kofa EW, Slutsky JR. Clinicians' attitudes to clinical practice guidelines: a systematic review. Med J Aust. 2002 Nov 4;177(9):502-6. PMID: 12405894. | Outcome |
| Ferron G, Martinez A, Gladieff L, Mery E, David I, Delannes M, Montastruc M, Balagué G, Picaud L, Querleu D. Adherence to guidelines in gynecologic cancer surgery. Int J Gynecol Cancer. 2014 Nov;24(9):1675-8. doi: 10.1097/IGC.0000000000000284. PMID: 25340292. | Outcome |
| Flodgren G, Conterno LO, Mayhew A, Omar O, Pereira CR, Shepperd S. Interventions to improve professional adherence to guidelines for prevention of device-related infections. Cochrane Database Syst Rev. 2013 Mar 28;(3):CD006559. doi: 10.1002/14651858.CD006559.pub2. PMID: 23543545. | Outcome |
| Flodgren G, Pomey MP, Taber SA, Eccles MP. Effectiveness of external inspection of compliance with standards in improving healthcare organisation behaviour, healthcare professional behaviour or patient outcomes. Cochrane Database Syst Rev. 2011 Nov 9;(11):CD008992. doi: 10.1002/14651858.CD008992.pub2. Update in: Cochrane Database Syst Rev. 2016 Dec 02;12 :CD008992. PMID: 22071861; PMCID: PMC4164461. | Outcome |
| Floor-Schreudering A, De Smet PA, Buurma H, Amini S, Bouvy ML. Clarity and applicability of drug-drug interaction management guidelines: a systematic appraisal by general practitioners and community pharmacists in the Netherlands. Drug Saf. 2011 Aug 1;34(8):683-90. doi: 10.2165/11587270-000000000-00000. PMID: 21751828. | Study design |
| Flottorp SA, Oxman AD, Krause J, Musila NR, Wensing M, Godycki-Cwirko M, Baker R, Eccles MP. A checklist for identifying determinants of practice: a systematic review and synthesis of frameworks and taxonomies of factors that prevent or enable improvements in healthcare professional practice. Implement Sci. 2013 Mar 23;8:35. doi: 10.1186/1748-5908-8-35. PMID: 23522377; PMCID: PMC3617095. | Outcome |
| Flu HC, Tamsma JT, Lindeman JH, Hamming JF, Lardenoye JH. A systematic review of implementation of established recommended secondary prevention measures in patients with PAOD. Eur J Vasc Endovasc Surg. 2010 Jan;39(1):70-86. doi: 10.1016/j.ejvs.2009.09.027. Epub 2009 Nov 11. PMID: 19910222. | Outcome |
| Fonhus MS, Dalsbo TK, Johansen M, Fretheim A, Skirbekk H, Flottorp SA. Patient‐mediated interventions to improve professional practice. Cochrane Database of Systematic Reviews 2018, Issue 9. Art. No.: CD012472. DOI: 10.1002/14651858.CD012472.pub2. | Outcome |
| Forrest JL, Miller SA. Evidence-based decision making in dental hygiene education, practice, and research. J Dent Hyg. 2001 Winter;75(1):50-63. PMID: 11314227. | Study design |
| Francke AL, Smit MC, de Veer AJ, Mistiaen P. Factors influencing the implementation of clinical guidelines for health care professionals: a systematic meta-review. BMC Med Inform Decis Mak. 2008 Sep 12;8:38. doi: 10.1186/1472-6947-8-38. PMID: 18789150; PMCID: PMC2551591. | Outcome |
| Freixa A, Moreira T, Bill O, Anani N. Implementability of stroke guidelines: a pragmatic comparison between US and European recommendations using eGLIA. Stud Health Technol Inform. 2015;210:256-60. PMID: 25991145. | Outcome |
| French CT, Diekemper RL, Irwin RS, Adams TM, Altman KW, Barker AF, Birring SS, Blackhall F, Bolser DC, Boulet LP, Braman SS, Brightling C, Callahan-Lyon P, Canning BJ, Chang AB, Coeytaux R, Cowley T, Davenport P, Diekemper RL, Ebihara S, El Solh AA, Escalante P, Feinstein A, Field SK, Fisher D, French CT, Gibson P, Gold P, Gould MK, Grant C, Harding SM, Harnden A, Hill AT, Irwin RS, Kahrilas PJ, Keogh KA, Lane AP, Lim K, Malesker MA, Mazzone P, Mazzone S, McCrory DC, McGarvey L, Molasiotis A, Murad MH, Newcombe P, Nguyen HQ, Oppenheimer J, Prezant D, Pringsheim T, Restrepo MI, Rosen M, Rubin B, Ryu JH, Smith J, Tarlo SM, Vertigan AE, Wang G, Weinberger M, Weir K; CHEST Expert Cough Panel. Assessment of Intervention Fidelity and Recommendations for Researchers Conducting Studies on the Diagnosis and Treatment of Chronic Cough in the Adult: CHEST Guideline and Expert Panel Report. Chest. 2015 Jul;148(1):32-54. doi: 10.1378/chest.15-0164. PMID: 25764280; PMCID: PMC4493878. | Outcome |
| Fullen BM, Baxter GD, O'Donovan BG, Doody C, Daly LE, Hurley DA. Factors impacting on doctors' management of acute low back pain: a systematic review. Eur J Pain. 2009 Oct;13(9):908-14. doi: 10.1016/j.ejpain.2008.11.013. Epub 2008 Dec 24. PMID: 19110456. | Outcome |
| Fung-Kee-Fung M, Kennedy EB, Biagi J, Colgan T, D'Souza D, Elit LM, Hunter A, Irish J, McLeod R, Rosen B. An organizational guideline for gynecologic oncology services. Int J Gynecol Cancer. 2015 May;25(4):551-8. doi: 10.1097/IGC.0000000000000400. PMID: 25756401. | Outcome |
| Gad El-Rab W, Zaïane OR, El-Hajj M. Formalizing clinical practice guideline for clinical decision support systems. Health Informatics J. 2017 Jun;23(2):146-156. doi: 10.1177/1460458216632272. Epub 2016 Mar 7. PMID: 26951569. | Study design |
| Gaebel W, Großimlinghaus I, Heun R, Janssen B, Johnson B, Kurimay T, Montellano P, Muijen M, Munk-Jorgensen P, Rössler W, Ruggeri M, Thornicroft G, Zielasek J; European Psychiatric Association. European Psychiatric Association (EPA) guidance on quality assurance in mental healthcare. Eur Psychiatry. 2015 Mar;30(3):360-87. doi: 10.1016/j.eurpsy.2015.01.011. Epub 2015 Feb 26. PMID: 25725593. | Outcome |
| Gagliardi AR, Brouwers MCDo guidelines offer implementation advice to target users? A systematic review of guideline applicabilityBMJ Open 2015;5:e007047. doi: 10.1136/bmjopen-2014-007047 | Outcome |
| Gagliardi AR, Dobrow MJ, Wright FC. How can we improve cancer care? A review of interprofessional collaboration models and their use in clinical management. Surg Oncol. 2011 Sep;20(3):146-54. doi: 10.1016/j.suronc.2011.06.004. Epub 2011 Jul 16. PMID: 21763127. | Outcome |
| García S, Martínez-Cengotitabengoa M, López-Zurbano S, Zorrilla I, López P, Vieta E, González-Pinto A. Adherence to Antipsychotic Medication in Bipolar Disorder and Schizophrenic Patients: A Systematic Review. J Clin Psychopharmacol. 2016 Aug;36(4):355-71. doi: 10.1097/JCP.0000000000000523. PMID: 27307187; PMCID: PMC4932152. | Outcome |
| Gethin G. Disseminating wound care guidelines: lessons from the literature. Br J Community Nurs. 2010 Sep;Suppl:S32-7. PMID: 21796844. | Study design |
| Gibson O, Lisy K, Davy C, Aromataris E, Kite E, Lockwood C, Riitano D, McBride K, Brown A. Enablers and barriers to the implementation of primary health care interventions for Indigenous people with chronic diseases: a systematic review. Implement Sci. 2015 May 22;10:71. doi: 10.1186/s13012-015-0261-x. PMID: 25998148; PMCID: PMC4465476. | Outcome |
| Gilbody S, Whitty P, Grimshaw J, Thomas R. Educational and organizational interventions to improve the management of depression in primary care: a systematic review. JAMA. 2003 Jun 18;289(23):3145-51. doi: 10.1001/jama.289.23.3145. PMID: 12813120. | Outcome |
| Girlanda F, Fiedler I, Ay E, Barbui C, Koesters M. Guideline implementation strategies for specialist mental healthcare. Curr Opin Psychiatry. 2013 Jul;26(4):369-75. doi: 10.1097/YCO.0b013e328361e7ae. PMID: 23673369. | Study design |
| Golbus JR, Wojcik BM, Charpie JR, Hirsch JC. Feeding complications in hypoplastic left heart syndrome after the Norwood procedure: a systematic review of the literature. Pediatr Cardiol. 2011 Apr;32(4):539-52. doi: 10.1007/s00246-011-9907-x. Epub 2011 Feb 20. PMID: 21336978. | Outcome |
| Gouvêa M, Novaes Cde O, Pereira DM, Iglesias AC. Adherence to guidelines for surgical antibiotic prophylaxis: a review. Braz J Infect Dis. 2015 Sep-Oct;19(5):517-24. doi: 10.1016/j.bjid.2015.06.004. Epub 2015 Aug 5. PMID: 26254691. | Outcome |
| Green SA, Bell D, Mays N. Identification of factors that support successful implementation of care bundles in the acute medical setting: a qualitative study. BMC Health Serv Res. 2017 Feb 7;17(1):120. doi: 10.1186/s12913-017-2070-1. PMID: 28173796; PMCID: PMC5297157. | Study design |
| Grimshaw JM, Shirran L, Thomas R, Mowatt G, Fraser C, Bero L, Grilli R, Harvey E, Oxman A, O'Brien MA. Changing provider behavior: an overview of systematic reviews of interventions. Med Care. 2001 Aug;39(8 Suppl 2):II2-45. PMID: 11583120. | Study design |
| Gunn J, Diggens J, Hegarty K, Blashki G. A systematic review of complex system interventions designed to increase recovery from depression in primary care. BMC Health Serv Res. 2006 Jul 16;6:88. doi: 10.1186/1472-6963-6-88. PMID: 16842629; PMCID: PMC1559684. | Outcome |
| Haines A, Kuruvilla S, Borchert M. Bridging the implementation gap between knowledge and action for health. Bull World Health Organ. 2004 Oct;82(10):724-31; discussion 732. PMID: 15643791; PMCID: PMC2623035. | Study design |
| Hale K, Capra S, Bauer J. Are nutrition messages lost in transmission? Assessing the quality and consistency of diabetes guideline recommendations on the delivery of nutrition therapy. Patient Educ Couns. 2016 Dec;99(12):1940-1946. doi: 10.1016/j.pec.2016.07.021. Epub 2016 Jul 21. PMID: 27473638. | Outcome |
| Hammond R; Taylor A. A NICE experience: the development of a multi-disciplinary guideline for the management of chronic heart failure.Physiotherapy Aug 2003;89(8):458-459. https://doi.org/10.1016/S0031-9406(05)60001-6 | Study design |
| Han, C.; Han, M. I.; Kwak, D. I. Barriers to implementation of practice guideline for depression in Korea. International Psychogeriatrics 2013;25():S154 | Study design |
| Harcombe Z, Baker JS, Cooper SM, et alEvidence from randomised controlled trials did not support the introduction of dietary fat guidelines in 1977 and 1983: a systematic review and meta-analysisOpen Heart 2015;2:e000196. doi: 10.1136/openhrt-2014-000196 | Outcome |
| Harrison A, Newell ML, Imrie J, Hoddinott G. HIV prevention for South African youth: which interventions work? A systematic review of current evidence. BMC Public Health 2010; 10: 102 | Outcome |
| Hewitt-Taylor J. Developing and using clinical guidelines. Nurs Stand. 2003 Oct 15-21;18(5):41-4. doi: 10.7748/ns2003.10.18.5.41.c3473. PMID: 14603766. | Study design |
| HFSA 2010 COMPREHENSIVE HEART FAILURE PRACTICE GUIDELINE\| Section 1: Development and Implementation of a Comprehensive Heart Failure Practice Guideline Heart Failure Society of America. VOLUME 16, ISSUE 6, E3-E33, JUNE 01, 2010. ttps://doi.org/10.1016/j.cardfail.2010.05.010 | Study design |
| Ilott I, Booth A, Rick J, Patterson M. How do nurses, midwives and health visitors contribute to protocol-based care? A synthesis of the UK literature. Int J Nurs Stud. 2010 Jun;47(6):770-80. doi: 10.1016/j.ijnurstu.2009.12.023. Epub 2010 Feb 18. PMID: 20170915. | Outcome |
| Jaramillo Y, Reznik M. Do United States' teachers know and adhere to the national guidelines on asthma management in the classroom? A systematic review. ScientificWorldJournal. 2015;2015:624828. doi: 10.1155/2015/624828. Epub 2015 Feb 2. PMID: 25729770; PMCID: PMC4333336. | Outcome |
| Kardas P, Lewek P, Matyjaszczyk M. Determinants of patient adherence: a review of systematic reviews. Front Pharmacol. 2013 Jul 25;4:91. doi: 10.3389/fphar.2013.00091. PMID: 23898295; PMCID: PMC3722478. | Outcome |
| Katbamna S, Baker R, Ahmad W, Bhakta P, Parker G. Development of guidelines to facilitate improved support of South Asian carers by primary health care teams. Qual Health Care. 2001 Sep;10(3):166-72. doi: 10.1136/qhc.0100166 PMID: 11533424; PMCID: PMC1743443. | Study design |
| Kilsdonk, E., Peute, L. W., & Jaspers, M. W. M. (2017). Factors influencing implementation success of guideline-based clinical decision support systems: A systematic review and gaps analysis. International Journal of Medical Informatics, 2017; 98, 56–64. doi:10.1016/j.ijmedinf.2016.12.001 | Outcome |
| Kirchner, H.; Fiene, M.; Ollenschläger, G. Assessment and implementation of guidelines.Rehabilitation 2003;42(2):74-82 | Study design |
| Köberlein, J., Vent, J., Mösges, R. et al. Effectiveness of guidelines in treatment of allergic rhinitis: an analysis of individual patient data. J Public Health 19, 563–568 (2011). https://doi.org/10.1007/s10389-011-0418-2 | Outcome |
| Lamontagne, Marie-Eve PhD; Gargaro, Judith BSc, MEd; Marier-Deschênes, Pascale BSW; Truchon, Catherine PhD, MSc Adm; Bayley, Mark Theodore MD; Marshall, Shawn MD, MSc, FRCPC; Kagan, Corinne BA, BPS Cert; Brière, Anabèle PhD; Swaine, Bonnie PhD A Survey of Perceived Implementation Gaps for a Clinical Practice Guideline for the Rehabilitation of Adults With Moderate to Severe Traumatic Brain Injury, Journal of Head Trauma Rehabilitation: September/October 2018 - Volume 33 - Issue 5 - p 306-316 doi: 10.1097/HTR.0000000000000430 | Outcome |
| Lau R, Stevenson F, Ong BN, et al. Achieving change in primary care—effectiveness of strategies for improving implementation of complex interventions: systematic review of reviewsBMJ Open 2015;5:e009993. doi: 10.1136/bmjopen-2015-009993 | Study design |
| Lazzerini M, Ciuch M, Rusconi S, Covi B. Facilitators and barriers to the effective implementation of the individual maternal near-miss case reviews in low/middle-income countries: a systematic review of qualitative studies. BMJ Open. 2018;8: e021281–e021281. doi:10.1136/bmjopen-2017-021281 | Outcome |
| Lee RC, Marshall D, Waddell C, Hailey D, Juzwishin D. Health technology assessment, research, and implementation within a health region in Alberta, Canada. Int J Technol Assess Health Care. 2003 Summer;19(3):513-20. doi: 10.1017/s0266462303000448. PMID: 12962337. | Outcome |
| Leentjens AF, Boenink AD, van der Feltz-Cornelis CM. Can we increase adherence to treatment recommendations of the consultation psychiatrist working in a general hospital? A systematic review. J Psychosom Res. 2010 Mar;68(3):303-9. doi: 10.1016/j.jpsychores.2009.07.006. PMID: 20159218. | Outcome |
| Leo CG, Mincarone P, Sabina S, Latini G, Wong JB. A conceptual framework for rationalized and standardized Universal Newborn Hearing Screening (UNHS) programs. Ital J Pediatr. 2016 Feb 12;42:15. doi: 10.1186/s13052-016-0223-1. PMID: 26872853; PMCID: PMC4751642. | Study design |
| Leone L, Pesce C. From delivery to adoption of physical activity guidelines: Realist synthesis. Int J Environ Res Public Health. CEVAS Center for Research and Evaluation, Rome, 00175, Italy; 2017;14. doi:10.3390/ijerph14101193 | Outcome |
| Levin A, Stevens LA. Executing change in the management of chronic kidney disease: perspectives on guidelines and practice. Med Clin North Am. 2005 May;89(3):701-9. doi: 10.1016/j.mcna.2004.11.005. PMID: 15755474. | Study design |
| Löffler C, Böhmer F. The effect of interventions aiming to optimise the prescription of antibiotics in dental care — A systematic review. PLoS One. Institute of General Practice, Rostock University Medical Center, Rostock, Germany; 2017;12. doi:10.1371/journal.pone.0188061 | Outcome |
| Madanat R, Mäkinen TJ, Aro HT, Bragdon C, Malchau H. Adherence of hip and knee arthroplasty studies to RSA standardization guidelines. A systematic review. Acta Orthop. 2014 Sep;85(5):447-55. doi: 10.3109/17453674.2014.934187. Epub 2014 Jun 23. PMID: 24954489; PMCID: PMC4164860. | Outcome |
| Mazières B, Thevenon A, Coudeyre E, Chevalier X, Revel M, Rannou F. Adherence to, and results of, physical therapy programs in patients with hip or knee osteoarthritis. Development of French clinical practice guidelines. Joint Bone Spine. 2008 Oct;75(5):589-96. doi: 10.1016/j.jbspin.2008.02.016. Epub 2008 Sep 19. PMID: 18805033. | Outcome |
| McAlister, F.A., Padwal, R. Implementation of Guidelines for Diagnosing and Treating Hypertension. Dis-Manage-Health-Outcomes 9, 361–369 (2001). https://doi.org/10.2165/00115677-200109070-00002 | Study design |
| McGuire DB. Barriers and strategies in implementation of oral care standards for cancer patients. Support Care Cancer. 2003 Jul;11(7):435-41. doi: 10.1007/s00520-003-0466-4. Epub 2003 Apr 12. PMID: 12692708. | Study design |
| Möhler R, Meyer G. Development methods of guidelines and documents with recommendations on physical restraint reduction in nursing homes: a systematic review. BMC Geriatr. 2015 Nov 21;15:152. doi: 10.1186/s12877-015-0150-9. PMID: 26589496; PMCID: PMC4654891. | Outcome |
| Morgano GP, Davoli M, Moja L, Amato L, Ferroni E, Tirani M. Guidelines 2.0: sviluppo sistematico di una checklist per la realizzazione di linee-guida affidabili [Guidelines 2.0: systematic development of a comprehensive checklist for a successful guideline enterprise]. Recenti Prog Med. 2015 Jun;106(6):249-79. Italian. doi: 10.1701/1884.20552. PMID: 26076414. | Study design |
| Moulding NT, Silagy CA, Weller DP. A framework for effective management of change in clinical practice: dissemination and implementation of clinical practice guidelines. Qual Health Care. 1999 Sep;8(3):177-83. doi: 10.1136/qshc.8.3.177. PMID: 10847875; PMCID: PMC2483658. | Study design |
| Moullin JC, Sabater-Hernández D, Fernandez-Llimos F, Benrimoj SI. A systematic review of implementation frameworks of innovations in healthcare and resulting generic implementation framework. Health Res Policy Syst. 2015 Mar 14;13:16. doi: 10.1186/s12961-015-0005-z. PMID: 25885055; PMCID: PMC4364490. | Outcome |
| Muñoz, L. Á S.; Cabeza, M. Á T.; Herrero, J. C.; Pérez, M. V. M.; Oñate, M. B. A.; Portugal, F. J. S.; Manchado, J. N. Experience in the development and implementation of a clinical pathway for community-acquired pneumonia in a district hospital. Revista de Calidad Asistencial 2006;21(6):299-310 | Study design |
| Murad MH. Clinical Practice Guidelines: A Primer on Development and Dissemination. Mayo Clin Proc. 2017 Mar;92(3):423-433. doi: 10.1016/j.mayocp.2017.01.001. PMID: 28259229. | Study design |
| Murphy EV. Clinical decision support: effectiveness in improving quality processes and clinical outcomes and factors that may influence success. Yale J Biol Med. 2014 Jun 6;87(2):187-97. PMID: 24910564; PMCID: PMC4031792. | Study design |
| Nagpal J, Sachdeva A, Sengupta Dhar R, Bhargava VL, Bhartia A. Widespread non-adherence to evidence-based maternity care guidelines: a population-based cluster randomised household survey. BJOG. 2015 Jan;122(2):238-47. doi: 10.1111/1471-0528.13054. Epub 2014 Aug 22. PMID: 25145674. | Study design |
| Nair M, Yoshida S, Lambrechts T, et alFacilitators and barriers to quality of care in maternal, newborn and child health: a global situational analysis through metareviewBMJ Open 2014;4:e004749. doi: 10.1136/bmjopen-2013-004749 | Outcome |
| Nasiri A, Balouchi A, Rezaie-Keikhaie K, Bouya S, Sheyback M, Rawajfah OA. Knowledge, attitude, practice, and clinical recommendation toward infection control and prevention standards among nurses: A systematic review. Am J Infect Control. Department of Anesthesiology, Emam Khomeini Hospital, Urmia University of Medical Sciences, Urmia, Iran; 2019;47: 827–833. doi:10.1016/j.ajic.2018.11.022 | Outcome |
| Neame MT, Chacko J, Surace AE, Sinha IP, Hawcutt DB. A systematic review of the effects of implementing clinical pathways supported by health information technologies. J Am Med INFORMATICS Assoc. 2019;26: 356–363. doi:10.1093/jamia/ocy176 | Outcome |
| Nielsen KK, Kapur A, Damm P, de Courten M, Bygbjerg IC. From screening to postpartum follow-up - the determinants and barriers for gestational diabetes mellitus (GDM) services, a systematic review. BMC Pregnancy Childbirth. 2014 Jan 22;14:41. doi: 10.1186/1471-2393-14-41. PMID: 24450389; PMCID: PMC3901889. | Outcome |
| Nilsen P, Aalto M, Bendtsen P, Seppä K. Effectiveness of strategies to implement brief alcohol intervention in primary healthcare. A systematic review. Scand J Prim Health Care. 2006 Mar;24(1):5-15. doi: 10.1080/02813430500475282. PMID: 16464809. | Outcome |
| Noonan VK, Wolfe DL, Thorogood NP, Park SE, Hsieh JT, Eng JJ; SCIRE Research Team. Knowledge translation and implementation in spinal cord injury: a systematic review. Spinal Cord. 2014 Aug;52(8):578-87. doi: 10.1038/sc.2014.62. Epub 2014 May 6. PMID: 24796445; PMCID: PMC4492721. | Study design |
| Oosthuysen J, Potgieter E, Blignaut E. Compliance with infection control recommendations in South African dental practices: a review of studies published between 1990 and 2007. Int Dent J. 2010 Jun;60(3):181-9. PMID: 20684444. | Outcome |
| O'Reilly P, Lee SH, O'Sullivan M, Cullen W, Kennedy C, MacFarlane A. Assessing the facilitators and barriers of interdisciplinary team working in primary care using normalisation process theory: An integrative review. PLoS One. 2017 May 18;12(5):e0177026. doi: 10.1371/journal.pone.0177026. Erratum in: PLoS One. 2017 Jul 24;12 (7):e0181893. PMID: 28545038; PMCID: PMC5436644. | Outcome |
| Pantoja T, Soto M. Guías de práctica clínica: una introducción a su elaboración e implementación [Clinical practice guidelines development and implementation: an introduction]. Rev Med Chil. 2014 Jan;142(1):98-104. Spanish. doi: 10.4067/S0034-98872014000100015. PMID: 24861121. | Study design |
| Paton F, Chambers D, Wilson P, et alEffectiveness and implementation of enhanced recovery after surgery programmes: a rapid evidence synthesisBMJ Open 2014;4:e005015. doi: 10.1136/bmjopen-2014-005015 | Outcome |
| Politi MC, Wolin KY, Légaré F. Implementing clinical practice guidelines about health promotion and disease prevention through shared decision making. J Gen Intern Med. 2013 Jun;28(6):838-44. doi: 10.1007/s11606-012-2321-0. Epub 2013 Jan 10. PMID: 23307397; PMCID: PMC3663950. | Study design |
| Price L, Macdonald J, Gozdzielewska L, Howe T, Flowers P, Shepherd L, et al. Interventions to improve healthcare workers’ hand hygiene compliance: A systematic review of systematic reviews. Infect Control Hosp Epidemiol. Safeguarding Health Through Infection Prevention Research Group, School of Health and Life Sciences, Glasgow Caledonian University, Cowcaddens Road, Glasgow, G4 0BA, United Kingdom; 2018;39: 1449–1456. doi:10.1017/ice.2018.262 | Outcome |
| Pucher PH, Johnston MJ, Aggarwal R, Arora S, Darzi A. Effectiveness of interventions to improve patient handover in surgery: A systematic review. Surgery. 2015 Jul;158(1):85-95. doi: 10.1016/j.surg.2015.02.017. Epub 2015 May 18. PMID: 25999255. | Outcome |
| Ramsey K. Review: Strategies to improve provider adoption and implementation of clinical practice guidelines were assessed. Ann Intern Med. 2017;166: JC58. doi:10.7326/ACPJC-2017-166-10-058 | Study design |
| Rockson SG. Appropriate secondary prevention of acute atherothrombotic events and strategies to improve guideline adherence. Postgrad Med. 2009 Jan;121(1):25-39. doi: 10.3810/pgm.2009.01.1952. PMID: 19179811. | Study design |
| Roen K, Arai L, Roberts H, Popay J. Extending systematic reviews to include evidence on implementation: methodological work on a review of community-based initiatives to prevent injuries. Soc Sci Med. 2006 Aug;63(4):1060-71. doi: 10.1016/j.socscimed.2006.02.013. Epub 2006 Mar 29. PMID: 16574289. | Study design |
| Ruiz-Pérez I, Rodríguez-Gómez M, Pastor-Moreno G, Escribá-Agüir V, Petrova D. Effectiveness of interventions to improve cancer treatment and follow-up care in socially disadvantaged groups. Psychooncology. Andalusian School of Public Health, Granada, Spain; 2019;28: 665–674. doi:10.1002/pon.5011 | Outcome |
| Sanduleanu S, Kaltenbach T, Barkun A, McCabe RP, Velayos F, Picco MF, Laine L, Soetikno R, McQuaid KR. A roadmap to the implementation of chromoendoscopy in inflammatory bowel disease colonoscopy surveillance practice. Gastrointest Endosc. 2016 Jan;83(1):213-22. doi: 10.1016/j.gie.2015.08.076. Epub 2015 Sep 11. PMID: 26364967. | Study design |
| Santesso N, Morgano GP, Jack SM, Haynes RB, Hill S, Treweek S, Schünemann HJ; DECIDE Workpackage 3 Group. Dissemination of Clinical Practice Guidelines: A Content Analysis of Patient Versions. Med Decis Making. 2016 Aug;36(6):692-702. doi: 10.1177/0272989X16644427. Epub 2016 Apr 18. PMID: 27091380. | Study design |
| Schwarz, P. Development and implementation of a European practice guideline and training standards for diabetes prevention - The IMAGE project - Implication for the Gulf states. Internist 2011;52():37-38. | Study design |
| Sehl J, O’Doherty J, O’Connor R, O’Sullivan B, O’Regan A. Adherence to COPD management guidelines in general practice? A review of the literature. Ir J Med Sci. University of Limerick Graduate Entry Medical School, Limerick, Ireland; 2018;187: 403–407. doi:10.1007/s11845-017-1651-7 | Outcome |
| Shiffman RN, Michel G, Essaihi A, Thornquist E. Bridging the guideline implementation gap: a systematic, document-centered approach to guideline implementation. J Am Med Inform Assoc. 2004 Sep-Oct;11(5):418-26. doi: 10.1197/jamia.M1444. Epub 2004 Jun 7. PMID: 15187061; PMCID: PMC516249. | Study design |
| Siering U, Eikermann M, Hausner E, Hoffmann-Eßer W, Neugebauer EA. Appraisal tools for clinical practice guidelines: a systematic review. PLoS One. 2013 Dec 9;8(12):e82915. doi: 10.1371/journal.pone.0082915. PMID: 24349397; PMCID: PMC3857289. | Outcome |
| Sinuff T, Cook D, Giacomini M, Heyland D, Dodek P. Facilitating clinician adherence to guidelines in the intensive care unit: A multicenter, qualitative study. Crit Care Med. 2007 Sep;35(9):2083-9. doi: 10.1097/01.ccm.0000281446.15342.74. PMID: 17855822. | Study design |
| Slade SC, Kent P, Patel S, Bucknall T, Buchbinder R. Barriers to Primary Care Clinician Adherence to Clinical Guidelines for the Management of Low Back Pain: A Systematic Review and Metasynthesis of Qualitative Studies. Clin J Pain. 2016 Sep;32(9):800-16. doi: 10.1097/AJP.0000000000000324. PMID: 26710217. | Outcome |
| Smith WR. Evidence for the effectiveness of techniques To change physician behavior. Chest. 2000 Aug;118(2 Suppl):8S-17S. doi: 10.1378/chest.118.2_suppl.8s. PMID: 10939994. | Study design |
| Sohn W, Ismail AI, Tellez M. Efficacy of educational interventions targeting primary care providers' practice behaviors: an overview of published systematic reviews. J Public Health Dent. 2004 Summer;64(3):164-72. doi: 10.1111/j.1752-7325.2004.tb02747.x. PMID: 15341140. | Study design |
| Solberg, L. I. Guideline implementation: what the literature doesn't tell us. Jt Comm J Qual Improv Sep 2000;26(9):525-37. https://doi.org/10.1016/S1070-3241(00)26044-6 | Study design |
| Song XP, Tian JH, Cui Q, Zhang TT, Yang KH, Ding GW. Could clinical pathways improve the quality of care in patients with gastrointestinal cancer? A meta-analysis. Asian Pac J Cancer Prev. 2014;15(19):8361-6. doi: 10.7314/apjcp.2014.15.19.8361. PMID: 25339029. | Outcome |
| Stetler CB, Damschroder LJ, Helfrich CD, Hagedorn HJ. A Guide for applying a revised version of the PARIHS framework for implementation. Implement Sci. 2011 Aug 30;6:99. doi: 10.1186/1748-5908-6-99. PMID: 21878092; PMCID: PMC3184083. | Study design |
| Suva G, Sharma T, Campbell KE, Sibbald RG, An D, Woo K. Strategies to support pressure injury best practices by the inter-professional team: A systematic review. Int Wound J. 2018;15: 580–589. doi:10.1111/iwj.12901 | Outcome |
| Thompson MA, Mugavero MJ, Amico KR, Cargill VA, Chang LW, Gross R, Orrell C, Altice FL, Bangsberg DR, Bartlett JG, Beckwith CG, Dowshen N, Gordon CM, Horn T, Kumar P, Scott JD, Stirratt MJ, Remien RH, Simoni JM, Nachega JB. Guidelines for improving entry into and retention in care and antiretroviral adherence for persons with HIV: evidence-based recommendations from an International Association of Physicians in AIDS Care panel. Ann Intern Med. 2012 Jun 5;156(11):817-33, W-284, W-285, W-286, W-287, W-288, W-289, W-290, W-291, W-292, W-293, W-294. doi: 10.7326/0003-4819-156-11-201206050-00419. Epub 2012 Mar 5. PMID: 22393036; PMCID: PMC4044043. | Outcome |
| Tibaldi G, Salvador-Carulla L, García-Gutierrez JC. From treatment adherence to advanced shared decision making: new professional strategies and attitudes in mental health care. Curr Clin Pharmacol. 2011 May;6(2):91-9. doi: 10.2174/157488411796151101. PMID: 21592062. | Study design |
| Townsend, L. How Effective are Interventions to Enhance Adherence to Psychiatric Medications? Practice Implications for Social Workers Working With Adults Diagnosed With Severe Mental Illness, Journal of Human Behavior in the Social Environment, 2009, 19:5, 512-530, DOI: 10.1080/10911350902987987 | Outcome |
| van Acker K, Léger P, Hartemann A, Chawla A, Siddiqui MK. Burden of diabetic foot disorders, guidelines for management and disparities in implementation in Europe: a systematic literature review. Diabetes Metab Res Rev. 2014 Nov;30(8):635-45. doi: 10.1002/dmrr.2523. PMID: 24470359. | Outcome |
| Van de Velde S, Heselmans A, Delvaux N, Brandt L, Marco-Ruiz L, Spitaels D, et al. A systematic review of trials evaluating success factors of interventions with computerised clinical decision support. Implement Sci. Norwegian Institute of Public Health, Centre for Informed Health Choices, Division for Health Services, Oslo, Norway; 2018;13. doi:10.1186/s13012-018-0790-1 | Outcome |
| Van de Velde S, Kunnamo I, Roshanov P, Kortteisto T, Aertgeerts B, Vandvik PO, et al. The GUIDES checklist: development of a tool to improve the successful use of guideline-based computerised clinical decision support. Implement Sci. 2018;13: 86. doi:10.1186/s13012-018-0772-3 | Outcome |
| Van de Werf F, Ardissino D, Bueno H, Collet JP, Gershlick A, Kolh P, Kristensen SD, Silber S, Verheugt F, Wojakowski W. Acute coronary syndromes: considerations for improved acceptance and implementation of management guidelines. Expert Rev Cardiovasc Ther. 2012 Apr;10(4):489-503. doi: 10.1586/erc.12.20. PMID: 22458581. | Study design |
| van Eikenhorst L, Taxis K, van Dijk L, de Gier H. Pharmacist-led self-management interventions to improve diabetes outcomes. A systematic literature review and meta-analysis. Front Pharmacol. Unit of PharmacoTherapy, -Epidemiology and -Economics, Groningen Research Institute of Pharmacy, University of Groningen, Groningen, Netherlands; 2017;8. doi:10.3389/fphar.2017.00891 | Outcome |
| Van Heuckelum M, Van Den Ende CHM, Houterman AEJ, Heemskerk CPM, Van Dulmen S, Van Den Bemt BJF. The effect of electronic monitoring feedback on medication adherence and clinical outcomes: A systematic review. PLoS One. Departments of Rheumatology and Pharmacy, Sint Maartenskliniek, Nijmegen, Netherlands; 2017;12. doi:10.1371/journal.pone.0185453 | Outcome |
| Vandenberg T, Coakley N, Nayler J, Degrasse C, Green E, Mackay JA, McLennan C, Smith A, Wilcock L, Trudeau ME. A framework for the organization and delivery of systemic treatment. Curr Oncol. 2009 Jan;16(1):4-15. doi: 10.3747/co.v16i1.297. PMID: 19229367; PMCID: PMC2644625. | Outcome |
| Vasilevska M, Ku J, Fisman DN. Factors associated with healthcare worker acceptance of vaccination: a systematic review and meta-analysis. Infect Control Hosp Epidemiol. 2014 Jun;35(6):699-708. doi: 10.1086/676427. Epub 2014 Apr 17. PMID: 24799647. | Outcome |
| Vernooij RW, Willson M, Gagliardi AR; members of the Guidelines International Network Implementation Working Group. Characterizing patient-oriented tools that could be packaged with guidelines to promote self-management and guideline adoption: a meta-review. Implement Sci. 2016 Apr 14;11:52. doi: 10.1186/s13012-016-0419-1. PMID: 27079375; PMCID: PMC4832541. | Outcome |
| Waters RE 2nd, Singh KP, Roe MT, Lotfi M, Sketch MH Jr, Mahaffey KW, Newby LK, Alexander JH, Harrington RA, Califf RM, Granger CB. Rationale and strategies for implementing community-based transfer protocols for primary percutaneous coronary intervention for acute ST-segment elevation myocardial infarction. J Am Coll Cardiol. 2004 Jun 16;43(12):2153-9. doi: 10.1016/j.jacc.2003.12.057. PMID: 15193673. | Study design |
| Weingart, S.N. Implementing practice guidelines: easier said than done. Isr J Health Policy Res 3, 20 (2014). https://doi.org/10.1186/2045-4015-3-20 | Study design |
| Wells S, Tamir O, Gray J, Naidoo D, Bekhit M, Goldmann D. Are quality improvement collaboratives effective? A systematic review. BMJ Qual Saf. Epidemiology and Biostatistics, School of Population Health, University of Auckland, Auckland, 1071, New Zealand; 2018;27: 226–240. doi:10.1136/bmjqs-2017-006926 | Outcome |
| Whellan DJ. Heart failure disease management: implementation and outcomes. Cardiol Rev. 2005 Sep-Oct;13(5):231-9. doi: 10.1097/01.crd.0000135765.60824.2f. PMID: 16106184. | Study design |
| Wilkinson, S. A.; McCray, S.; Beckmann, M.; Parry, A.; McIntyre, H. D. Barriers and enablers to translating gestational diabetes guidelines into practice. Practical Diabetes 2014;31(2):67-72a. | Study design |
| Yang C, Hao Z, Yu D, Xu Q, Zhang L. The prevalence rates of medication adherence and factors influencing adherence to antiepileptic drugs in children with epilepsy: A systematic review and meta analysis. Epilepsy Res. Department of Pharmacy, Evidence-based Pharmacy Center, West China second hospital, Sichuan University, Key Laboratory of Birth Defects and Related Diseases of Women and Children (Sichuan University), Ministry of Education, China; 2018;142: 88–99. doi:10.1016/j.eplepsyres.2018.03.018 | Outcome |
