# Supplementary material for: Strategies for the implementation of clinical practice guidelines in public health: an overview of systematic reviews
Source: Health Res Policy Syst. 2022 Jan 24;20:13. doi: 10.1186/s12961-022-00815-4 (PMC8785489; doi:10.1186/s12961-022-00815-4)
Supplement: Supplementary file 3 — Additional file 3. Characteristics and AMSTAR2 of the systematic reviews. [file 12961_2022_815_MOESM3_ESM.pdf]

Additional file 3 - Characteristics and AMSTAR2 of the systematic reviews

Author	Objectives	Study designs included and number	Clinical area	Country / setting	Target of implementation strategies	Methodological quality of included studies
AL Zoubi 2018	To summarize and evaluate evidence about the effectiveness of knowledge translation (KT) interventions to improve the uptake and application of clinical practice guidelines and best practices for a wide range of musculoskeletal (MSK) disorders and health care professionals.	11 unique studies reported in 13 publications:<br>6 individual RCTs<br>5 cluster RCTs	Musculoskeletal (MSK) disorders	Netherlands (n = 4)<br>UK (n = 2)<br>Australia (n = 2)<br>USA (n = 1)<br>Switzerland (n = 1)<br>Ireland (n = 1)	Organisational or professional	Yes<br>Criteria developed by the Cochrane Handbook for Risk of Bias Assessment (version 5.1.0)
Bauer 2002	To locate and review all published peer-reviewed reports that provide quantitative information on rates of adherence to specific mental health guidelines.	41 studies<br>26 cross-sectional studies, 6 pre/post studies, 9 controlled trials: 9 (only theses studies reported the effectiveness of the strategies)	Mental health	USA,UK, Australia, Scotland, France, Canada<br>Primary-care or other medical clinics, medical centers, nursing homes, acute medical Ward, community-based sampling	Healthcare workers	No
Bighelli2016	To examine the efficacy of guideline implementation strategies in improving process outcomes (performance of healthcare providers) and patient outcomes and to explore which components of different guideline implementation strategies could influence them.	6 RCTs	Schizophrenia-spectrum disorders	Denmark, German, USA, UK - Psychiatric units, hospitals	Healthcare workers	Yes<br>Risk of bias (Cochrane handbook)

Brusamento 2012	To evaluate the effectiveness of strategies to implement clinical guidelines for chronic disease management in primary care in EU Member States.	21 studies<br>14 cluster-RCTs, 3 RCTs, 3 controlled-before-and-after design, 1 controlled clinical trial	- Osteoarthritis<br>- COPD<br>- Hypertension<br>- Coronary heart disease<br>- Asthma<br>- Diabetes mellitus	EU Member States<br>Finland, France, Germany, Italy, Spain, Sweden, UK, Denmark, Netherlands<br>- Primary care level	Healthcare workers	Yes<br>Risk of bias based on the tools described in the Cochrane Handbook for Systematic Reviews of Interventions
Car 2019	To evaluate the effectiveness of digital education in improving the adoption of clinical practice guidelines.	17 studies<br>14 RTCs<br>3 cluster-RCTs	Resuscitation, paediatric basic life support, diabetes, preventive care, labour management, myocardial infarction management, hypertension management, physical therapy, prostate cancer screening, dementia, urinary tract infection and heart failure	Except for one study from an upper middle-income country, all studies were from high-income countries with ten studies from the USA	Healthcare workers	Yes<br>Cochrane Risk of Bias Tool
Chaillet 2006	To evaluate what strategies effectively implement clinical practice guidelines in obstetric care, and to identify barriers to change and facilitators in obstetrics.	33 studies<br>10 cluster RCTs, 6 RCTs, 1 controlled before-and-after study, 16 interrupted time series studies	Obstetric care	Low, middle and high resource settings<br>Obstetric care	Health and non-health workers and patients	Yes<br>Quality was assessed using the Cochrane Effective Practice and Organisation of Care criteria. For controlled trials, broadly, these criteria evaluate selection bias, characteristics of study and control providers, exclusion bias, follow-up of patients, detection bias, baseline performance, reliability of first outcomes, and

						protection against contamination. For interrupted time series, the criteria evaluate protection against secular changes, appropriate data analysis, reason for the number of points pre- and post-intervention, specification of the shape of the intervention effect, protection against detection bias, completeness of data set, and reliable first outcome (details of specific criteria were not reported).
Cortoos 2007	To provide a classification of used Community-acquired pneumonia (CAP)-guideline implementation interventions and to quantify the impact of different interventions and their intensity of use on several processes of care, clinical and/or economic outcomes.	27 studies (30 papers)<br>14 retrospective before-after, 2 prospective before-after, 4 retrospective controlled before-and-after study, 2 RCTs, 1 cluster-RCT, 4 interrupted time series	Pneumonia	USA, UK, Canada, Australia<br>Hospital setting	Healthcare professionals	Yes<br>Possible sources of systematic errors are selection bias (systematic differences in comparison groups), performance bias (systematic differences in care provided apart from the intervention being evaluated), attrition bias (systematic differences in withdrawals from the trial) and detection bias (systematic differences in outcome assessment).
Damiani 2010	To asses the impact of computerized clinical guidelines (CCG) on the process of care compared with non-computerized clinical guidelines.	45 studies<br>39 experimental studies, 6	Acute and chronic	Europe, USA, Oceania<br>Academic and non-academic centres	Healthcare professionals	Yes<br>Score assessing five potential sources of study bias

		observational studies				
De Angelis 2016	To identify research on health professionals' perceived usability and practice behavior change of information and communication technologies for the dissemination of clinical practice guidelines.	21 studies<br>20 RCT, 1 NRCT	NR	United States, Canada, Europe, and 1 international study conducted in 63 countries	Healthcare professionals	Yes<br>Cochrane risk of bias tool
Dexheimer 2014	To determine the most prevalent method of guideline implementation (paper, computer-generated, or computerized), as reported in the literature; establish which methods significantly improved clinical care; and identify the factors most commonly associated with a successful and sustainable asthma guideline implementation.	104 (101 articles)<br>61 pre-post design, 56 prospective design, 27 population based case series, 23 consecutive case series, 13 randomized trials, 15 non-blinded trials, 16 nonconsecutive case series double-blinded trials(5)<br>Best-case series (6)	Asthma	USA, UK, Canada, Australia, Netherlands, Singapore, New Zeland, Brazil, Saudi Arabia, Germany, France, Oman, Switzerland, Italy, Iran, Japan, Taiwan, Korea, Thailand, and the United Arab Emirates<br>- Academic institutions, non-academic institutions and 3 did not describe the setting.<br>- Studies looked at outpatients, emergency department and inpatients, patients in other settings (e.g., the home).	Healthcare workers and patients	Yes<br>Methodology of Wang et al., which grades study design on a 5-point scale with Level 1 studies being the most scientifically rigorous and Level 5 studies having a more lenient study design [21].
Diehl 2016	To study was to systematically review the effects of interventions to improve the implementation of guidelines in nursing homes.	5 cluster-randomised controlled trials	- Oral health care<br>- Reduction of physical restraints<br>- Atypical antispychotic prescribing<br>- Urinary tract	United States, Germany, Netherland, Australia, Belgium<br>Nursing homes	Healthcare workers	Yes (GRADE)<br>- The overall quality of the studies for the results in the category professional practice was rated low and very low<br>- The overall quality of the

			infection<br>- Falls best practice strategies<br>- Pressure ulcer			studies for the results in the category patient outcome was rated low and very low
Docherty 2017	To examine studies of the effectiveness of evidence-based CPG implementation across physical and mental health care, to inform mental healthcare provision in low and middle income countries (LMICs), and to identify transferable lessons from other non-communicable diseases to mental health.	6 studies<br>5 cluster randomised controlled trials (cluster RCTs), 1 uncontrolled before and after study	- Mental health care: anxiety and panic disorder<br>- Physical health: acute coronary syndromes, diabetes mellitus, asthma, COPD, and hypertension	Low or middle income country<br>Brazil, China, Thailand, Nepal, South Africa and Egypt<br>- General hospitals in urban areas<br>- Primary health care centres (urban area, rural areas, and mix of urban and rural areas)	Healthcare workers	Yes<br>Risk of bias assessments
Donnellan 2013	To review the literature on adherence to clinical guidelines including the impact adherence has on achieving quality indicators for stroke care and to identify actual and perceived supports and hindrances for adherence to best practice.	27 studies (Interventions to improve adherence to generic stroke guidelines studies n = 7); Studies that included interventions to improve adherence to specific stroke guidelines (n = 4)<br>7 prospective observational studies, 7 retrospective audits, 6 quantitative surveys, 3 quasi-experimental	Stroke care	USA, Australia, UK, Scotland<br>National, regional, local and clinical institution level	Healthcare organisations, healthcare workers	No

		studies, 1 qualitative interviews using focus groups				
Flodgren 2016	To evaluate the effectiveness of implementation tools developed and disseminated by guideline producers, which accompany or follow the publication of a CPG, to promote uptake. A secondary objective is to determine which approaches to guideline implementation are most effective.	4 cluster-RCTs	- Non-specific low back pain<br>- Hospital-acquired pneumonia<br>- Patients who required a thyroid-function test<br>- Diabetes<br>- Diabetes and high risk of cardiovascular disease	Netherlands, France, USA and Canada<br>- Private physiotherapy practices<br>- Hospitals<br>- Family	Healthcare workers	Yes<br>Cochrane's 'Risk of bias' tool
Grimshaw 2004	The aims of the study were:<br>- to undertake a systematic review of the effectiveness and costs of different guideline development, dissemination and implementation strategies;<br>- to estimate the resource implications of different development, dissemination and implementation strategies.<br>- to develop a framework for deciding when it is efficient to develop and introduce clinical guidelines based upon the potential costs and benefits of the targeted clinical activity and the effectiveness and costs of guideline development and introduction.	235<br>110 C-RCTs, 29 P-RCTs (P-RCTs individual randomisation at the level of the patient), 7 C-CCTs (cluster alocation-controlled clinical trials), 10 P-CCT (controlled clinical trials at the level of the patient) , 40 controlled before and after studies, 39 interrupted time series	NR	- Most in USA, UK, Canada, Australia and Netherlands<br>- The remaining studies were conducted in Denmark, France, Germany, Israel, Mexico, New Zealand Norway, Sweden and Thailand.<br>- Primary care (39%), followed by inpatient settings (19%) and generalist outpatient or ambulatory care settings (although all of these studies were undertaken in the USA and may be equivalent to primary care) (19%).<br>- Thirty-six (15%) studies	Healthcare workers and patients	Yes<br>Cochrane EPOC group's methodological quality criteria

				were based within mixed settings, either inpatient and outpatient, at the interface between settings, or a mix of communityand hospital-based care.<br>- The remaining studies were set in nursing homes or long-term care facilities (3%), emergency departments (2%), specialist outpatient care (1%) and a military medical clinic (0.4%). The setting was not clear from the reports of three (1%) studies.		
Gross et al. 2001	To determine the best methods for changing prescribing practices and to facilitate better implementation of these guidelines.	40 studies<br>Study design not shown	Antimicrobial Usage	Not reported	Healthcare workers	No
Häggman-Laitila 2017	To systematically review the literature on the outcomes of educational interventions relevant to nurses with regard to guideline implementation.	13 studies based on a quase-experimental study design	- Paediatric inpatient units<br>- Healthy eating habits<br>- Venipuncture practices<br>- Central line care<br>- Asthma prevention<br>- Patient education<br>- Pain assessment and management<br>- Diabetes foot ulcer assessment<br>- Venous	USA, Australia, Iran, Singapure<br>- Nursing units	Healthcare workers	Yes<br>- The quality of the quasi - experimental study with a comparison group was evaluated in accordance with the quality assessment tool of Thomas et al (2004) using the format and dictionary published on the website of the Effective Public Health Practice Project (www.ephpp.ca).<br>- The quality of the

			thromboembolism prevention<br>- Insulin infusion and glycaemic control in cardiac surgery<br>- Cervical cancer screening in adolescents<br>- Pain assessment and management<br>- Breastfeeding<br>- Neonatal abstinence syndrome<br>- Using hyaluronidase to treat intravenous extravasations			uncontrolled studies using a before - and after design was appraised with a tool published on the website of the National Heart, Lung, and Blood Institution (www.nhlbi.nih.gov).
Hakkennes 2008	To evaluate the effects of the introduction of clinical guidelines for allied health professionals, and to estimate the effectiveness of the guideline dissemination and implementation strategies used.	14 studies<br>12 RCTs, 1 non-randomised controlled clinical, 1 non-randomised controlled before and after	NR	USA, Australia, Netherlands, UK<br>- Clinical institution level, provider level	Healthcare organisations, healthcare workers	Yes<br>Cochrane EPOC Review Group with each criteria scored as done, not clear or not done
Heselmans 2009	To systematically and comprehensively search the literature for studies evaluating the effectiveness of computer-based guideline implementation systems in ambulatory care settings with the multidimensionality of the guideline (the guideline needed to consist of several aspects or steps) and real-time interaction with the system during	27 studies<br>20 cluster-RCTs, 1 controlled clinical trials, two controlled before and after studies, 4 interrupted time series	- Chronic and acute diseases (Angina, coronary artery disease, heart failure and ischemic heart disease, hypertension, dyslipidemia, hypercholesterolemia, diabetes, asthma/ chronic obstructive pulmonary disease	USA, four in the UK, five in the Netherlands, two in Norway, one in France, and one in Finland.<br>Ambulatory care of which four were performed in the emergency department.	Healthcare professionals	Yes<br>EPOC data collection checklists

	consultation as important inclusion criteria.		(COPD), depression, HIV, occupational exposure to body fluids, acute low back pain, otitis media, fever in children, common pediatric diseases).<br>- Tobacco use cessation.			
Imamura 2017	To assess whether strategies to promote the use of guidelines can improve obstetric practices in low- and middle-income countries (LMICs).	9 studies<br>8 RCT, 1 controlled before and after studies	Obstetric care	Georgia, Syria, China and Pakistan, Senegal, Mali, Benin, Malawi, Mexico, Thailand, Argentina, Guatemala, India, Pakistan, Kenya, and Zambia<br>Health centers and hospitals, health facilities	Healthcare professionals	Yes<br>Nine criteria specified by EPOC; Risk of bias was incorporated into the assessment of the level of evidence using the GRADE approach.
Jeffery 2015	To synthesize evidence about the effectiveness of interventions that target healthcare providers to improve adherence to cardiovascular disease guidelines and patient outcomes.	38 studies<br>36 cluster-RTC, 2 RCT	Cardiovascular	Eighteen studies took place in the USA, 14 were completed in Europe (the Netherlands, Italy, England, and Norway), two took place in Canada, one in South Africa, one in Brazil, one in Asia-Pacific area, and one in the Virgin Islands.	Healthcare professionals	Yes<br>Cochrane Risk of Bias tool for RCTs
Jordan 2017	To conduct a systematic review on implementation strategies for clinical practice guidelines in the intensive care unit. Knowledge gained from this review might assist critical care practitioners to use implementation strategies for the	8 RCT	Critical ill (Quality of care improvement, nutritional support, prevention of nosocomial infections, palliative care,	Canada, Australia, England and Taiwan, and USA<br>- Adult ICUs and neonatal ICUs	Healthcare organisations	Yes<br>Joanna Briggs Institute Meta Analysis of Statistics Assessment and Review Instrument (JBI-MAStARI) Critical Appraisal Tool for

	use of clinical practice guidelines in the intensive care units (ICUs).		reduction of medical errors in the intensive care unit)			Randomized Controlled Trials and Experimental studies
Liang 2017	To summarize current research in the field of guideline implementation to describe if and how theory has been used to plan or evaluate the implementation and use of guidelines among physicians, who are frequently the target users of guidelines.	42 studies, 8 studies evaluated the impact of interventions:<br>2 Single cohort study (before-after), 4 Randomized controlled trial, 2 qualitative study embedded in a randomized controlled trial	Treatment and self-management of osteoarthritis<br>Venous thromboembolism prevention<br>Management of acute musculoskeletal pain<br>Prescribing, management of sore throat<br>Prevention, detection, evaluation, and treatment of high blood pressure<br>Smoking cessation<br>Episiotomy and management of the third stage of labor<br>Tuberculosis screening	Studies were conducted in the United Kingdom (10, 23.8%), Australia (9, 21.4%), the United States (7, 16.7%), the Netherlands (6, 14.3%), Canada (3, 7.1%), Iran (3, 7.1%), Argentina (1, 2.4%), Belgium (1, 2.4%), Germany (1, 2.4%), and Saudi Arabia (1, 2.4%).	Organisational or professional Health care settings (e.g., hospitals, ambulatory clinics, community-based physician offices)	No
Lineker 2010	To evaluate the influence of educational programs to implement Clinical Practice Guidelines (CPG) for osteoarthritis (OA) and rheumatoid arthritis (RA) in primary care.	7 studies<br>6 randomized controlled trials, 1 controlled before and after study	Osteoarthritis (OA) and rheumatoid arthritis (RA)	NR<br>Primary care environment	Healthcare professionals	Yes<br>Critical review form — Quantitative studies. McMaster University: Hamilton
Liu 2012	To examine what strategies may improve adherence to Pelvic inflammatory disease (PID) diagnosis and management guidelines	3 studies<br>2 RCT, 1 interrupted time series	Pelvic inflammatory disease	USA<br>Hospital and outpatient facilities<br>- A single paediatric	Healthcare workers and patients	NR

				outpatient department<br>- 5 hospital emergency departments or paediatric and adolescent medicine clinics<br>- Members of the section on Emergency Medicine of the American Academy of Paediatrics		
Medves 2010	To synthesis the literature relevant to guideline dissemination and implementation strategies for healthcare teams and team-based practice.	89 studies<br>28 RCTs, 34 descriptive/case series and 27 comparable cohort studies	Not reported	The 88 studies were predominately from the USA (53.4%) and the UK (11.4%), with a further eight Canadian and four Australian studies. Other countries are represented by three or less studies. The total number of countries was 17.	Healthcare workers, patients, healthcare organisations, structural interventions	MASTARI - ferramenta de avaliação crítica
Nguyen 2018	To determine whether interventions targeted at healthcare professionals are effective to enhance prescribing and health outcomes in patients with ischaemic heart diseases.	13 studies<br>4 RCT, 9 cluster-RCT	Cardiology	11 out of 13 studies were performed in North America and Europe	Organisational or professional	Yes<br>Grading of Recommendation, Assessment, Development, and Evaluation (GRADE)
Okelo 2013	To assess whether interventions targeting health care providers improve adherence to asthma care guidelines and subsequently improve outcomes. Health care process outcomes were considered, such as patients receiving appropriate treatment, and clinical outcomes, such as hospitalizations.	68 studies reported in 73 articles	Asthma	Not reported<br>Clinical institution level, provider level	Healthcare organisations, healthcare workers	Yes<br>Cochrane Collaboration's tool for assessing risk of bias.

Shanbhag 2018	Accordingly, the primary objective of the review was to examine the effectiveness of implementation interventions in increasing physician adherence to the specified HF guideline recommendations. Secondary objectives were to assess the effect of implementation interventions on clinical outcomes, and to identify process and contextual factors that influence implementation success.	38 studies<br>10 were RCT, 3 controlled before-after studies, 2 interrupted time series studies, 18 uncontrolled before-after studies, 4 retrospective cohort design, 1 combination of retrospective and prospective cohort designs	Cardiology	A majority of the studies were conducted in the USA (n=26), and the remainder in Europe (n=10) and Australia (n=2).<br>Sixteen studies were conducted in inpatient settings, twenty-one in outpatient settings and one involved care in both settings.	Health care settings (inpatient settings, outpatient settings and both settings)	Yes<br>EPOC Data Collection Checklist to evaluate RCTs, cluster RCTs, controlled before-after studies and interrupted time series studies.<br>For cluster RCTs, additional criteria of recruitment bias, loss of cluster and incorrect analysis according to the Cochrane Handbook for Systematic Reviews of Interventions was used.<br>For cohort studies, the Cochrane Collaboration's tool to assess risk of bias in cohort studies was used.<br>For uncontrolled before-after studies, the National Institute of Health's quality assessment tool for before-after studies with no control group was used.
Thomas 1998	(1) To identify evaluations (in any setting) of clinical practice guidelines and of related dissemination and implementation strategies in nursing, midwifery, health visiting, and other professions allied to medicine—podiatry, speech and language therapy, physiotherapy, occupational therapy, dietetics, clinical psychology, pharmacy, and radiography.	18 studies<br>3 randomised controlled trials explicitly evaluated guideline dissemination or implementation strategies	- Pneumococcal and influenza vaccinations<br>- Use and follow up of medications<br>- Urinary catheter care	Ambulatory medical practices	Healthcare workers	Yes

	(2) To derive estimates of the eVectiveness and cost eVectiveness of clinical practice guidelines as strategies for promoting improved professional practice and enhanced patient outcomes in nursing and professions allied to medicine.					
Tooher 2003	To search the literature for studies about effectiveness of strategies in implementing evidence-based guidelines and recommendations for the prevention and/or management of pressure ulcers and the characteristics of sustainable implementation of these strategies.	20 studies<br>Study designs not shown	Pressure ulcer	USA, German, UK, Australia<br>Hospitals, nursing homes, community care centres, residential homes, universities, tertiary care hospital, long-term care setting, regional health-care system	Healthcare organisations, healthcare workers, patients	No
Unverzagt 2014	To understand and compare different implementation strategies concerning guidelines targeting primary or secondary prevention and treatment of cardiovascular diseases.	52 studies<br>39 cluster-RCT, 19 RCT	Cardiovascular disease	Health systems differed between low-regulated health systems (Canada and USA) where 29 trials were conducted, and 22 trials were conducted in European countries with a higher regulation (Belgium, Germany, Denmark, Great Britain, Italy, Netherland, Sweden, Switzerland and Spain). Three further trials were conducted in Israel, Pakistan and Taiwan.	Healthcare workers	The internal validity of eligible trials according to the Cochrane Collaboration risk of bias tool.
van der Wees 2008	To answer the question: What is the effectiveness of strategies to increase the implementation of physiotherapy clinical guidelines?	5 articles/3 cluster-RCT	Physiotherapy clinical - low back pain guidelines	Australia, Netherlands, UK<br>Institutional and provider level (Physiotherapists)	Healthcare organisations, healthcare workers	Yes<br>Checklist adapted from the EPOC Group data collection checklist

Watkins 2015	To synthesise the literature on implementation of clinical guidelines to community pharmacy.	22 studies<br>10 RCTs, 3 non-randomised controlled trials, 1 controlled before and after studies and 8 quasi-experimental or observational studies	- Proton pump inhibitors (National Prescribing Service proton pump inhibitor dosage recommendation)<br>- Non-prescription drugs<br>- Gynaecology and Obstetrics<br>- Diabetes<br>- Methotrexate<br>- Asthma<br>- Medicines and driving<br>- Smoking cessation<br>- Osteoporosis<br>- Guideline-based counselling<br>- Ketorolac<br>- Statins drugs<br>- Good pharmacy practice	Australia, USA UK,Netherlands, Belgium, Canada, Finland, Germany, and Switzerland<br>- Community pharmacy setting	Healthcare workers	Yes<br>Studies that were either randomised controlled trials (RCTs), non-randomised controlled trials (NRCTs) or controlled before and after studies (CBAs) were evaluated for bias using the EPOC risk of bias tool. Other studies in this review were cohort or quasi-experimental studies and were evaluated for quality using the Newcastle-Ottawa quality assessment scale for cohort studies.
Weinmann 2007	To summarize the evidence pertaining to benefits of mental health guidelines in the view of specific implementation strategies.	18 studies<br>9 RCT, 6 nonrandomized-controlled clinical trials (CCT), 3 before-after studies without contemporary control group	Psychiatric	The majority of studies were conducted in the USA (twelve studies) followed by the UK (four studies), Denmark and Canada (one study each).<br>- Primary care<br>- General hospital<br>- Specialist mental healthcare settings<br>- Mental health clinicians of	Healthcare organisations, healthcare workers	Yes<br>Methodological quality was assessed by using the Cochrane Effective Practice and Organisation of Care Review Group (EPOC) criteria as reported by Grimshaw (10). To apply EPOC criteria, we sought the method of concealment allocation (only for RCTs), data on baseline

				a managed behavioural healthcare organization		measurement, follow-up of professionals and patients, blinded outcome assessment, reliability of primary outcome measures, protection against contamination and further characteristics of the study designs according to EPOC. Compliance with methodological criteria were judged by using an established checklist (http://www.epoc.uottawa.ca/checklist2002.doc).
Wensing 1998	To evaluate the effectiveness of interventions in influencing the implementation of guidelines and adoption of innovations in general practice.	143 studies (61 were selected for the analysis of the effectiveness of the interventions)<br>39 randomized controlled trials, 22 controlled before and after studies	NR	United States<br>United Kingdom<br>Canada<br>Several other countries	Professional Interventions,<br>Financial Interventions,<br>Organizational Interventions	Yes

NR: not reported

Reference	AMSTAR	Q1	Q2	Q3	Q4	Q5	Q6	Q7	Q8	Q9	Q10	Q11	Q12	Q13	Q14	Q15	Q16	Overall methodology quality
AL Zoubi 2018	10	Yes	No	Yes	Yes	Yes	Yes	Yes	Yes	Yes	No	NM	NM	Yes	No	NM	Yes	Low
Bauer 2002	3	NA	No	Yes	Yes	No	No	No	Yes	No	No	NM	NM	No	?	NM	No	Critically low
Bighelli2016	8	Yes	No	Yes	No	Yes	Yes	No	No	Yes	No	Yes	No	Yes	No	No	Yes	Critically low
Brusamento 2012	10	Yes	No	Yes	Yes	Yes	Yes	Yes	Yes	Yes	No	NM	NM	Yes	No	NM	Yes	Low
Car 2019	12	Yes	Yes	Yes	Yes	Yes	Yes	No	Yes	Yes	No	Yes	No	Yes	Yes	No	Yes	Critically low
Chaillet 2006	6	Yes	No	Yes	Yes	No	Yes	No	No	Yes	No	NM	NM	Yes	No	NM	No	Critically low
Cortoos 2007	9	Yes	No	Yes	Yes	Yes	Yes	No	Yes	No	No	NM	NM	Yes	Yes	NM	Yes	Critically low
Damiani 2010	8	Yes	No	Yes	Yes	Yes	Yes	No	Yes	Yes	No	NM	NM	No	No	NM	Yes	Critically low
De Angelis 2016	10	Yes	No	Yes	Yes	Yes	Yes	Yes	Yes	Yes	No	NM	NM	Yes	No	NM	Yes	Low
Dexheimer 2014	7	Yes	No	Yes	Yes	Yes	No	No	Yes	Yes	No	NM	NM	No	No	NM	Yes	Critically low
Diehl 2016	11	Yes	Yes	Yes	Yes	Yes	No	Yes	Yes	Yes	No	NM	NM	Yes	No	NM	Yes	Moderate
Docherty 2017	10	Yes	No	Yes	Yes	Yes	Yes	Yes	Yes	Yes	No	NM	NM	Yes	No	NM	Yes	Low
Donnellan 2013	6	Yes	No	Yes	Yes	Yes	No	No	Yes	No	No	NM	NM	No	No	NM	Yes	Critically low
Flodgren 2016	10	Yes	No	Yes	Yes	Yes	Yes	Yes	Yes	Yes	No	NM	NM	Yes	Yes	NM	No	Low
Grimshaw 2004	7	Yes	No	Yes	Yes	No	Yes	No	No	Yes	No	NM	NM	Yes	Yes	NM	No	Low
Gross et al. 2001	3	Yes	No	No	Yes	No	no	No	No	No	No	NM	NM	Yes	No	NM	No	Critically low
Häggman-Laitila 2017	7	Yes	No	Yes	Yes	Yes	No	No	Yes	Yes	No	NM	NM	No	No	NM	Yes	Critically low
Hakkennes 2008	7	Yes	No	Yes	Yes	Yes	No	No	Yes	Yes	No	NM	NM	No	No	NM	Yes	Critically low
Heselmans 2009	10	Yes	No	Yes	Yes	Yes	Yes	yes	Yes	Yes	No	NM	NM	Yes	No	NM	Yes	Low
Imamura 2017	10	Yes	Yes	Yes	Yes	Yes	Yes	No	Yes	Yes	No	NM	NM	Yes	No	NM	Yes	Low
Jeffery 2015	13	Yes	Yes	Yes	Yes	Yes	Yes	No	Yes	Yes	No	Yes	Yes	Yes	Yes	NM	Yes	Low
Jordan 2017	6	Yes	Yes	No	Yes	Yes	Yes	No	Yes	No	No	NM	NM	No	No	NM	No	Critically low
Liang 2017	7	Yes	No	Yes	Yes	Yes	Yes	No	Yes	No	No	NM	NM	No	No	NM	Yes	Critically low
Lineker 2010	5	Yes	No	Yes	Yes	No	No	No	Yes	Yes	No	NM	NM	No	No	NM	No	Critically low
Liu 2012	4	Yes	No	Yes	Yes	No	No	No	Yes	No	No	NM	NM	No	No	NM	No	Critically low
Medves 2010	5	Yes	No	No	Yes	Yes	Yes	No	No	No	No	NM	NM	No	No	NM	Yes	Critically low
Nguyen 2018	14	Yes	Yes	Yes	Yes	Yes	Yes	No	Yes	Yes	Yes	Yes	Yes	Yes	Yes	Yes	No	Low
Okelo 2013	9	Yes	Yes	Yes	Yes	Yes	Yes	No	Yes	No	No	NM	NM	No	No	Yes	Yes	Critically low

Shanbhag 2018	11	Yes	Yes	Yes	Yes	Yes	Yes	No	Yes	Yes	No	NM	NM	Yes	Yes	NM	Yes	Low
Thomas 1998	7	Yes	No	Yes	Yes	Yes	Yes	No	Yes	No	No	NM	NM	No	Yes	NM	No	Critically low
Tooher 2003	4	Yes	No	Yes	Yes	No	No	No	Yes	No	No	NM	NM	No	No	NM	No	Critically low
Unverzagt 2014	12	Yes	No	Yes	Yes	Yes	Yes	No	Yes	Yes	No	Yes	Yes	Yes	Yes	No	Yes	Critically low
van der Wees 2008	6	Yes	No	Yes	Yes	Yes	No	No	Yes	Yes	No	NM	NM	No	No	NM	No	Critically low
Watkins 2015	10	Yes	Yes	Yes	Yes	Yes	Yes	No	Yes	Yes	No	NM	NM	Yes	No	NM	Yes	Low
Weinmann 2007	6	Yes	No	Yes	Yes	Yes	No	No	Yes	No	No	NM	NM	No	No	NM	Yes	Critically low
Wensing 1998	2	No	No	Yes	No	Yes	No	No	No	No	No	NM	NM	No	No	NM	No	Critically low

NM: Non metanalysis
