# Supplementary material for: Strategies for the implementation of clinical practice guidelines in public health: an overview of systematic reviews
Source: Health Res Policy Syst. 2022 Jan 24;20:13. doi: 10.1186/s12961-022-00815-4 (PMC8785489; doi:10.1186/s12961-022-00815-4)
Supplement: Supplementary file 4 — Additional file 4. Effectiveness of guideline implementation strategies from systematic reviews by type of outcome. [file 12961_2022_815_MOESM4_ESM.docx]

Additional file 4 - Effectiveness of guideline implementation strategies from systematic reviews by type of outcome.

| **Outcome**  **Strategy** | **Process** | | | | **Professional** | | | | **Patient** | | | | **Economic** | |
| --- | --- | --- | --- | --- | --- | --- | --- | --- | --- | --- | --- | --- | --- | --- |
|  | **SS** | | **MS** | | **SS** | | **MS** | | **SS** | | **MS** | | **MS** | |
|  | **NoA** | **E** | **NoA** | **E** | **NoA** | **E** | **NoA** | **E** | **NoA** | **E** | **NoA** | **E** | **NoA** | **E** |
| Reminders | 385 | 59% | 234 | 28% | 0 | 0% | 17 | 65% | 26 | 15% | 93 | 33% | 12 | 17% |
| Educational materials | 59 | 25% | 366 | 30% | 14 | 36% | 23 | 61% | 34 | 56% | 141 | 21% | 20 | 20% |
| Educational meetings | 50 | 34% | 346 | 27% | 20 | 70% | 48 | 58% | 15 | 0% | 137 | 19% | 18 | 11% |
| Audit and feedback | 25 | 36% | 226 | 36% | 0 | 0% | 19 | 68% | 0 | 0% | 89 | 24% | 5 | 40% |
| Clinical multidisciplinary teams | 20 | 0% | 17 | 6% | 0 | 0% | 9 | 56% | 4 | 0% | 11 | 36% | 1 | 0% |
| Local opinion leaders | 20 | 45% | 69 | 48% | 0 | 0% | 4 | 100% | 2 | 0% | 28 | 21% | 2 | 0% |
| C*are pathways* | 15 | 80% | 6 | 83% | 0 | 0% | 0 | 0% | 11 | 0% | 5 | 20% | 1 | 0% |
| Practice support | 12 | 58% | 30 | 63% | 0 | 0% | 3 | 100% | 1 | 0% | 29 | 24% | 1 | 0% |
| Academic detailing | 10 | 30% | 151 | 29% | 0 | 0% | 5 | 40% | 3 | 0% | 61 | 16% | 10 | 20% |
| Continuous quality improvement | 9 | 56% | 1 | 100% | 3 | 0% | 0 | 0% | 5 | 20% | 3 | 100% | 0 | 0% |
| Financial interventions | 7 | 0% | 21 | 52% | 0 | 0% | 0 | 0% | 3 | 0% | 3 | 0% | 0 | 0% |
| Patient-directed interventions | 7 | 0% | 18 | 33% | 0 | 0% | 0 | 0% | 8 | 50% | 20 | 15% |  | 0% |
| Continuity of care | 3 | 67% | 29 | 10% | 0 | 0% | 0 | 0% | 0 | 0% | 9 | 0% | 1 | 0% |
| Organisational culture | 3 | 0% | 73 | 19% | 0 | 0% | 12 | 83% | 0 | 0% | 36 | 14% | 0 | 0% |
| Tailored interventions | 1 | 100% | 4 | 50% | 0 | 0% | 0 | 0% | 1 | 0% | 1 | 0% | 0 | 0% |
| Structural intervention | 1 | 0% | 46 | 2% | 0 | 0% | 0 | 0% | 0 | 0% | 12 | 0% | 1 | 0% |
| Monitoring the performance of the delivery of healthcare | 1 | 100% | 26 | 35% | 0 | 0% | 0 | 0% | 0 | 0% | 0 | 0% | 6 | 0% |
| Communities of practice | 0 | 0% | 6 | 33% | 0 | 0% | 0 | 0% | 0 | 0% | 0 | 0% | 0 | 0% |
| Case management | 0 | 0% | 9 | 67% | 0 | 0% | 1 | 100% | 0 | 0% | 9 | 44% | 1 | 0% |
| Communication between providers | 0 | 0% | 3 | 67% | 0 | 0% | 3 | 33% | 0 | 0% | 0 | 0% | 0 | 0% |
| Patient incentives | 0 | 0% | 6 | 67% | 0 | 0% | 0 | 0% | 0 | 0% | 7 | 29% | 1 | 0% |
| Education intervention | 0 | 0% | 6 | 83% | 0 | 0% | 0 | 0% | 0 | 0% | 4 | 75% | 0 | 0% |
| Patient-mediated Intervention | 0 | 0% | 54 | 0% | 0 | 0% | 0 | 0% | 0 | 0% | 15 | 0% | 0 | 0% |
| Educational games | 0 | 0% | 0 | 0% | 2 | 100% |  | 0% | 0 | 0% | 0 | 0% | 0 | 0% |
| Marketing | 0 | 0% | 0 | 0% | 0 | 0% | 0 | 0% | 0 | 0% | 0 | 0% | 0 | 0% |
| Mass media | 0 | 0% | 9 | 22% | 0 | 0% | 0 | 0% | 0 | 0% | 0 | 0% | 6 | 0% |
| Community mobilization | 0 | 0% | 9 | 0% | 0 | 0% | 0 | 0% | 0 | 0% | 13 | 8% | 0 | 0% |
| Local consensus processes | 0 | 0% | 26 | 19% | 0 | 0% | 0 | 0% | 0 | 0% | 12 | 25% | 0 | 0% |
| Administrative restriction | 0 | 0% | 3 | 0% | 0 | 0% | 0 | 0% | 0 | 0% | 5 | 0% | 1 | 0% |
| Information and communication technology | 0 | 0% | 20 | 60% | 0 | 0% | 6 | 67% | 0 | 0% | 9 | 22% | 3 | 0% |
| **TOTAL** | **628** | **51%** | **1814** | **29,44%** | **39** | **54%** | **150** | **64%** | **113** | **28%** | **752** | **22%** | **90** | **13%** |

E: proportion of statistically positive results compared to the total of analyzes performed; NoA: number of number of comparisons analyzed; Single strategy; MS: Multifaceted Strategy.
